# Analysis of HubP-dependent cell pole protein targeting in *Vibrio cholerae* uncovers novel motility regulators

**DOI:** 10.1371/journal.pgen.1009991

**Published:** 2022-01-12

**Authors:** Ipek Altinoglu, Guillaume Abriat, Alexis Carreaux, Lucía Torres-Sánchez, Mickaël Poidevin, Petya Violinova Krasteva, Yoshiharu Yamaichi

**Affiliations:** 1 Université Paris-Saclay, CEA, CNRS, Institute for Integrative Biology of the Cell (I2BC), Gif-sur-Yvette, France; 2 Graduate School of Structure and Dynamics of Living Systems, Université Paris-Saclay, Orsay, France; 3 Doctoral School of Therapeutic Innovation ITFA. Université Paris-Saclay, Orsay, France; 4 ‘Structural Biology of Biofilms’ Group, European Institute of Chemistry and Biology (IECB), Pessac, France; 5 Université de Bordeaux, CNRS, Bordeaux INP, CBMN, UMR 5248, Pessac, France; Max-Planck-Institute for Marine Microbiology: Max-Planck-Institut fur marine Mikrobiologie, GERMANY

## Abstract

In rod-shaped bacteria, the emergence and maintenance of long-axis cell polarity is involved in key cellular processes such as cell cycle, division, environmental sensing and flagellar motility among others. Many bacteria achieve cell pole differentiation through the use of polar landmark proteins acting as scaffolds for the recruitment of functional macromolecular assemblies. In *Vibrio cholerae* a large membrane-tethered protein, HubP, specifically interacts with proteins involved in chromosome segregation, chemotaxis and flagellar biosynthesis. Here we used comparative proteomics, genetic and imaging approaches to identify additional HubP partners and demonstrate that at least six more proteins are subject to HubP-dependent polar localization. These include a cell-wall remodeling enzyme (DacB), a likely chemotaxis sensory protein (HlyB), two presumably cytosolic proteins of unknown function (VC1210 and VC1380) and two membrane-bound proteins, named here MotV and MotW, that exhibit distinct effects on chemotactic motility. We show that while both Δ*motW* and Δ*motV* mutants retain monotrichous flagellation, they present significant to severe motility defects when grown in soft agar. Video-tracking experiments further reveal that Δ*motV* cells can swim in liquid environments but are unable to tumble or penetrate a semisolid matrix, whereas a *motW* deletion affects both tumbling frequency and swimming speed. Motility suppressors and gene co-occurrence analyses reveal co-evolutionary linkages between MotV, a subset of non-canonical CheV proteins and flagellar C-ring components FliG and FliM, whereas MotW regulatory inputs appear to intersect with specific c-di-GMP signaling pathways. Together, these results reveal an ever more versatile role for the landmark cell pole organizer HubP and identify novel mechanisms of motility regulation.

## Author summary

Cell polarity is the result of controlled asymmetric distribution of protein macrocomplexes, genetic material, membrane lipids and cellular metabolites, and can play crucial physiological roles not only in multicellular organisms but also in unicellular bacteria. In the opportunistic cholera pathogen *Vibrio cholerae*, the polar landmark protein HubP tethers key actors in chromosome segregation, chemotaxis and flagellar biosynthesis and thus converts the cell pole into an important functional microdomain for cell proliferation, environmental sensing and adaptation between free-living and pathogenic life-styles. Using a comparative proteomics approach, we here-in present a comprehensive analysis of HubP-dependent cell pole protein sorting and identify novel HubP partners including ones likely involved in cell wall remodeling (DacB), chemotaxis (HlyB) and motility regulation (MotV and MotW). Unlike previous studies which have identified early roles for HubP in flagellar assembly, functional, genetic and phylogenetic analyses of its MotV and MotW partners suggest a direct role in flagellar rotary mechanics and provide new insights into the coevolution and functional interdependence of chemotactic signaling, bacterial motility and biofilm formation.

## Introduction

Bacterial cells contain subcellular domains in which dedicated protein machineries localize to secure key cellular functions such as cell division, motility, chemotaxis, chromosome segregation, morphological differentiation and virulence [1–4]. Two apparent subcellular compartments in rod-shaped bacteria are the midcell and cell poles. Their functional differentiation is subject to dynamic spatiotemporal control, which is perhaps best exemplified by the fact that cell division by binary fission happens at the midcell, yet leads to the creation of new cell poles in the daughter cells. Compared to the regulatory processes controlling cell division ([5,6] for review), the molecular mechanisms determining cell pole maturation and function remain to be elucidated. Whereas protein localization patterns can be highly dynamic depending on cell cycle stages or environmental stimuli, some proteins exhibit propensity to remain stably localized at one or two poles by harnessing a variety of underlying mechanisms such as sensing of negative membrane curvature, nucleoid occlusion, diffusion and capture by self-polymerization, or interaction with specific lipids or protein partners [7]. These include the so-called polar landmark proteins which not only associate with the cell pole(s) but also serve to recruit specific binding partners for the assembly of functional macromolecular complexes [7–9].

To date, several and diverse polar landmark proteins have been identified across the bacterial world, including DivIVA in Gram-positive firmicutes and actinomycetes, and PopZ and TipN proteins in α-proteobacteria such as *Caulobacter crescentus* ([7,10] for review). In the cholera pathogen *Vibrio cholerae*, a large ~180 kDa membrane protein–HubP–has been shown to organize polar identity [11]. The bacterium is known to have a multipartite genome (chromosome 1 (chr1) with 3.0 Mb and chromosome 2 (chr2) with 1.1 Mb) and be highly motile with monotrichous flagellation. HubP has been shown to modulate the localization of the chr1 replication origin region by directly interacting with the ParA1 chromosome partitioning protein, involved in chromosome segregation [11]. Moreover, HubP promotes polar localization of both chemotaxis array proteins, through interactions with the ParC/ParP complex, and monotrichous flagellum assembly through interactions with the FlhG and SflA regulators [11–15]. HubP has a multidomain architecture comprising a LysM peptidoglycan-binding domain in the periplasm, coiled-coil dimerization regions on both sides of a single transmembrane helix, a large and highly acidic cytosolic domain containing multiple aspartate and glutamate-rich repeats and a C-terminal tetratricopeptide repeat (TPR) region. Interestingly, while the periplasmic LysM domain is critical for its polar localization, characterized HubP protein partners are known to interact primarily with HubP’s highly acidic cytosolic modules [11].

Although characterized polar landmark proteins and their respective binding partners feature both structural and functional diversity, the organization of the chromosome segregation, chemotaxis and flagellar motility machineries at the cell poles represents a recurrent theme across the bacterial domain of life. The bacterial flagellum consists of three parts, the basal body (motor), the filament (propeller) and the hook (a structural linker between the two) and its rotary dynamics are directly coupled with chemotactic signaling ([16,17] for review). Counter-clockwise rotation of the motor generates force for the bacteria to move forward, whereas a transient switch to clockwise rotation, controlled by chemotactic signaling inputs to the flagellar rotor, or C-ring, induces cell tumbling and re-orientation in swimming direction. In *Vibrio* species, chemotaxis signaling proteins cluster at the cell poles [18], similar to the flagellar apparatus. Membrane-bound methyl-accepting chemotaxis proteins (MCPs) form large hexagonal arrays in the inner membrane, transducing different environmental stimuli to downstream chemotaxis proteins. CheW couplers link the MCP sensors to CheA kinases, upon which the latter activate and transfer phosphoryl groups to CheY response regulators. Phospho-CheY proteins directly bind the flagellar C-ring components FliM and FliN to induce a temporary counterclockwise-to-clockwise switch in rotation which reorients the cell’s swimming direction. In addition to canonical chemotaxis signaling proteins, some bacteria–including *V*. *cholerae*–harbor hybrid, multi-domain proteins such as the CheV (CheW-CheY) fusions [19]. The *V*. *cholerae* genome encodes multiple paralogues of the various chemotaxis proteins, with most genes found in 3 main clusters. Cluster II has been shown to be essential for chemotactic behavior under normal cell growth conditions [20,21], and it also encodes the HubP partners ParC/ParP known to recruit certain CheA, CheW and CheY paralogues [22,23].

To unveil a more comprehensive picture of the *Vibrio* cell pole organization, we sought to identify further interaction partners of HubP. To this end, we took advantage of minicells, that are small spherical cells lacking chromosomal DNA. In contrast to membrane vesicles that bud from the outer membrane, minicells are derived from asymmetrical cell divisions near the cell pole. In *Escherichia coli*, mutations in the *min* locus results in minicell generation [24,25] and this property has been used as a tool for the identification of pole-targeted *E*. *coli* proteins by comparative analyses of the minicell versus rod cell membrane proteomes [26]. Our previous study, however, showed that *min* mutants of *V*. *cholerae* do not bud minicells off their poles due to distinctive subcellular organization of their chromosomal DNA [27]. Presumably, the nucleoid occlusion mechanism which antagonizes FtsZ ring formation over the nucleoid [28,29] would prevent cell division near the cell pole in *V*. *cholerae*, where the chromosome 1 origin is tethered. Indeed, we showed that when a Δ*minD* mutation is combined with secondary mutations to release the chr1 origin from the cell pole (Δ*hubP*, Δ*parA1* or Δ*parS1*), the resulting double mutants indeed produce *V*. *cholerae* minicells [27].

By comparative proteomics on minicells derived from HubP^+^ and HubP^-^ *V*. *cholerae* cells, we thus characterized HubP-specific cell pole protein targeting and, together with genetic and imaging assays, demonstrate HubP-dependent polar localization for six different proteins, including a putative chemotaxis protein (VCA0220), a peptidoglycan modifying enzyme (VC0632), two small, likely cytosolic proteins of unknown function (VC1210 and VC1380) and two inner membrane-tethered partners (VC1909 and VC2232). As opposed to previous characterized HubP partners that bind the polar landmark through the latter’s cytosolic modules, we show that in two of the newly identified proteins–VC0632 and VC2232 –polar targeting appears dependent exclusively on periplasmic modules. We further examined putative involvement of the newly identified HubP interaction partners in *Vibrio* motility, and show that the hypothetical VC1909 and VC2232 proteins (named here MotV and MotW, respectively) are both positive regulators of flagellar motility that exhibit strong co-evolutionary and functional links with both chemotaxis and flagellar C-ring components.

## Results

### Minicells as a model of cell pole microcompartments for the identification of HubP-dependent protein targeting to the pole

**Fig 1:**
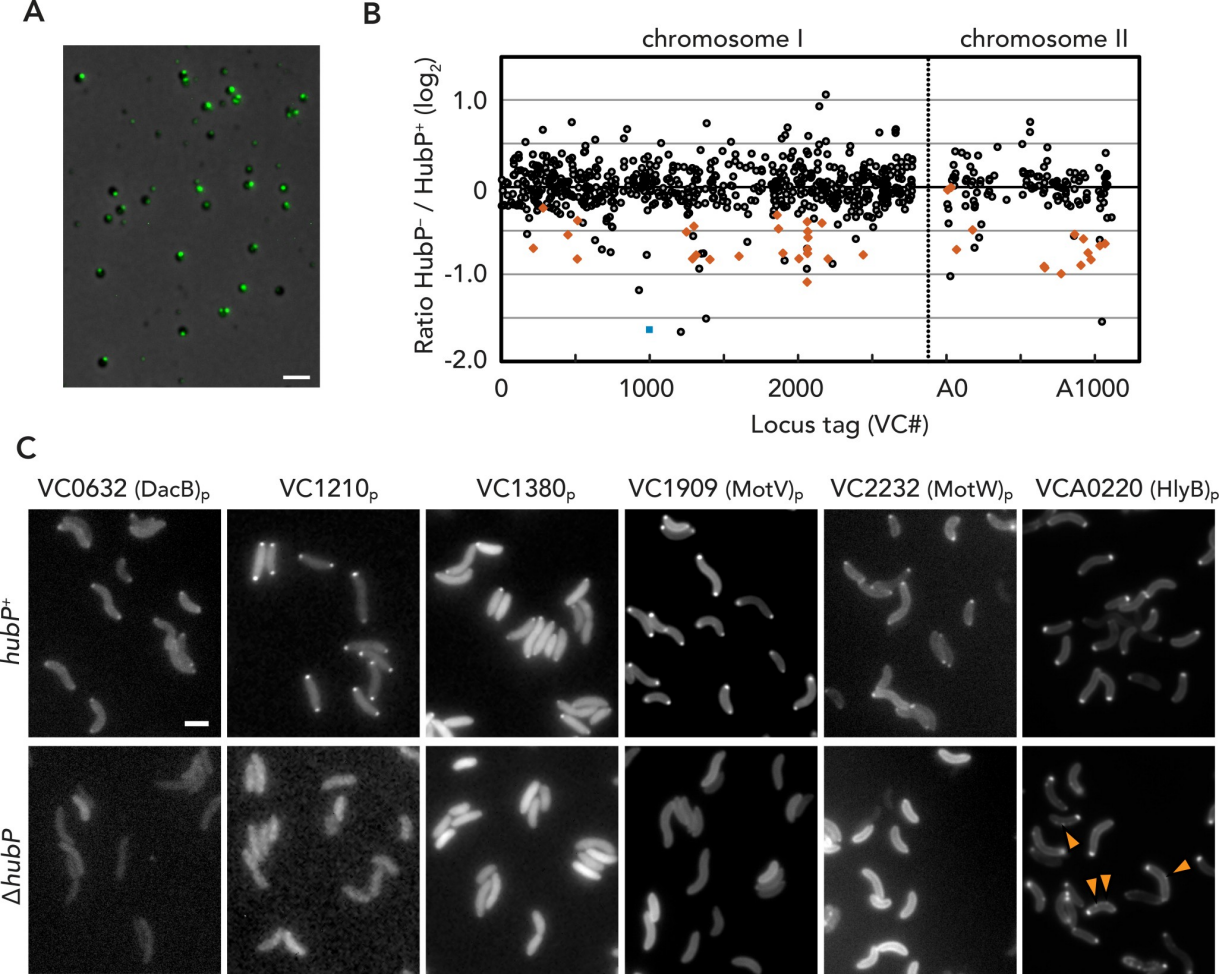
Comparative proteomics of minicells in search of polar proteins. (A) Representative fluorescent microscopy image of minicells purified from YBB2143 *(ΔminD ΔparA1 hubP*::*hubP-yfp*). YFP signals are pseudocolored in green and merged with the corresponding phase contrast image. (B) Summary plot of iTRAQ results. Horizontal axis: proteins’ locus tag on each chromosome; Y-axis, log2 of HubP^-^/HubP^+^ value from two independent experiments. A blue square indicates HubP (VC0998); known proteins related to chemotaxis are indicated by orange diamonds. (C) Representative fluorescence microscopy images of C-terminal GFP fusions of indicated candidate proteins in *hubP*^*+*^ and *ΔhubP V*. *cholerae* cells. Bars = 2 μm.

**Fig 2:**
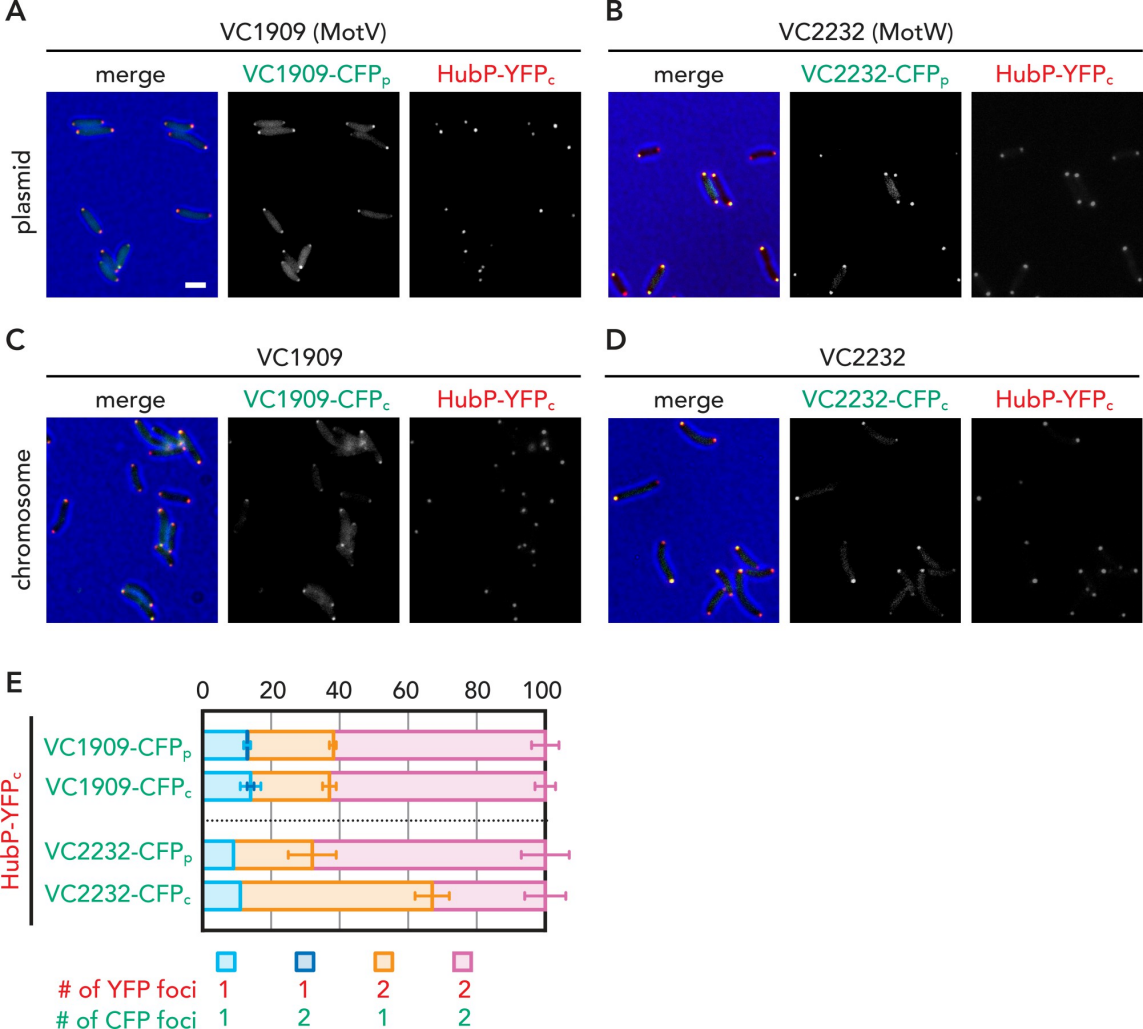
Bipolarity of polar proteins. (A-D) Representative fluorescent microscopy images of cells expressing indicated fluorescent protein fusions expressed from either plasmid (_p_) or native chromosomal locus (referred as _c_). (E) Fraction of cells (%) exhibiting uni- (1) or bi- (2) polar foci. Average and standard deviations of 2 independent analyses (each counted >1000 cells) are shown.

To confirm that *V*. *cholerae* minicells well represent cell pole microcompartments, we introduced *hubP-yfp* under native promoter in the *ΔminD ΔparA1* background and purified minicells by sequential centrifugations for microscopic observation. As shown in Fig 1A, the purified minicells showed discrete fluorescent foci, suggesting that they indeed represent cell pole compartments and likely retain a native pole architecture apart from the disrupted interactions specific to the ParABS1 partitioning system (due to *ΔparA1*). Therefore, we proceeded to prepare minicells from HubP^+^ (*ΔminD ΔparA1*) and HubP^-^ (*ΔminD ΔparA1 ΔhubP*) strains for the quantitative identification of HubP-dependent changes in the minicell proteomes. We used the iTRAQ (isobaric Tags for Relative and Absolute Quantitation [30]) tandem mass spectrometry method to differentially label and simultaneously determine and compare the amounts of proteins derived from purified HubP^+^ and HubP^-^ *V*. *cholerae* minicells. From a total of ~ 800 identified proteins, ~ 50 were found to be enriched in the HubP^+^ background. Confirming the validity of the assay, they include expected polar proteins such as FlhG and ParC, as well as ~30 downstream chemotaxis proteins including various MCPs (Fig 1B and S1 Table). We then examined the subcellular localization of the 21 remaining proteins by generating plasmids for their ectopic expression as fusion proteins carrying C-terminal monomeric superfolder GFP (hereafter GFP) that were expressed in HubP^+^ and Δ*hubP* backgrounds. Among them, 12 protein fusions showed diffuse cellular fluorescence and three were found to form inclusion bodies as visible under phase contrast (S1A and S1B Fig). Six proteins–VC0632, VC1210, VC1380, VC1909, VC2232 and VCA0220 –exhibited polar fluorescent foci confirming their localized enrichment at the poles (Figs 1C, 2 and S1C). Importantly, the polar localization of these GFP fusions was severely disrupted in a Δ*hubP* background with VCA0220 forming additional non-polar foci, similar to ParC distribution in Δ*hubP* cells (see below), while other proteins exhibited diffuse fluorescence with or without stronger signal at the cell periphery (Fig 1C). Conversely, upon co-expression of the plasmid-based fusions with fluorescent HubP variants (plasmid-based HubP-PAmCherry or chromosomal HubP-YFP expression), all six proteins showed their fluorescence foci co-localizing with the HubP signal (Figs 2 and S1C). Together, these data not only corroborate that the six proteins are specifically targeted to the cell poles in *V*. *cholerae* but also that their polar localization is dependent on direct or indirect interactions with the polar landmark protein HubP.

### Predicted topologies and function of identified HubP partners

Previous genome-wide assessments of essential genes in *V*. *cholerae* suggest that all six polar proteins identified as HubP partners here are dispensable for the viability and normal growth of the bacterium [31,32]. Indeed, we successfully constructed clean deletion mutants for each individual gene and none of them showed significant defects in culture growth (LB, S2A Fig) or irregular cell shape under microscopic observation (S2B Fig). Furthermore, plasmid-based expression of HubP-GFP led to the formation of polar fluorescent foci in all mutant backgrounds, suggesting that none of the six proteins act upstream of HubP in cell pole targeting (S2C Fig). Predicted protein folds, oligomeric states, topology and domain architectures for the four proteins are shown in S3 Fig.

### VCA0220

VCA0220 has been previously annotated as hemolysin secretion protein HlyB due to indirect evidence of its involvement in secretion of the cytotoxic HlyA hemolysin encoded by an adjacent gene [33]. The protein is not to be confused, however, with homologs of the HlyB ATPase binding cassette (ABC) transporters of *E*. *coli* or *Bordetella pertussis* that are responsible for hemolysin secretion by the assembly of a HlyB-HlyD-TolC Type I secretion system ([34] for review). Lines of evidence, including sequence and fold prediction analyses, reveal VCA0220 as a classical chemotaxis transducer protein with a periplasmic Tar/Tsr ligand binding module and intracellular HAMP and methyl-accepting domains in tandem ([35,36]; S3A and S3B Fig), suggesting that any potential roles in hemolysin secretion are likely indirect. Indeed, the subcellular localization pattern of the protein in Δ*hubP* cells is very similar to that of ParC and downstream chemotaxis proteins (Fig 1C; [11]). In addition, we show here that HlyB is often displaced in a Δ*parC* background (S1D Fig), which further indicates that the protein belongs to the MCP superfamily of chemotaxis array-forming methyl-accepting signal transducers [36].

### VC0632

VC0632, hereafter DacB_Vc_, is a conserved homolog of the *E*. *coli* DacB protein (DacB_Ec_), also known as penicillin-binding protein 4 (PBP4) [37]. DacB homologs are bifunctional, homodimeric periplasmic proteins with known molecular architecture and peptidoglycan DD-endopeptidase and DD-carboxypeptidase activities (S3A and S3C Fig). Although DacB proteins are not essential for peptidoglycan biogenesis and cell growth neither in *E*. *coli*, nor *V*. *cholerae*, they have been proposed to cooperate with other cell wall modifying enzymes in envelope maturation and maintenance [37–39]. Indeed, the protein’s HubP-dependent targeting to the pole, as well as HubP’s own dependence on peptidoglycan binding for polar localization [11] support a model of cell pole-specific cell wall remodeling mechanisms.

### Hypothetical proteins VC1210 and VC1380

VC1210 is annotated as a hypothetical, 31 kDa protein of unknown function and homologs of it are found only in a subset of *Vibrio* species. The subcellular localization of the VC1210-GFP fusion in a Δ*hubP* background suggests that it is expressed in the cytosol, which is consistent with the lack of a detectable N-terminal signal peptide or other motifs for protein secretion (Figs 1C and S3D). Fold prediction analyses suggested significant structural similarity to aminoglycoside nucleotidyl transferases that are involved in the development of resistance against aminoglycoside antibiotics, however, the Δ*vc1210* mutant did not show significant difference in sensitivity to kanamycin or gentamycin and the protein’s function at the pole remains unknown (S3E Fig). Hypothetical protein VC1380 is a short, 116 amino acid-long polypeptide which does not feature detectable conserved domains, transmembrane segments or secretion signal motifs (S3F Fig). Consistent with the localization pattern of the VC1380-GFP fusion in Δ*hubP* cells, the protein is indeed likely produced in the cytosol (Fig 1C), however its specific function remains to be further examined.

### VC1909

VC1909 is annotated as a hypothetical protein with unknown function and is predicted to have a single N-proximal transmembrane helix leaving the remaining ~ 230 C-terminal amino acids in the cytosol (S3A and S3G Fig). Interestingly, VC1909 is a homolog of the *Aliivibrio* (formerly *Vibrio*) *fischeri* protein VF1491, which was identified as a putative motility regulator in soft-agar motility assays on an *A*. *fischeri* transposon mutant collection [40]. In addition, a recent study reported that VC1909/VF1491 homologs co-localize and interact with HubP in *Shewanella putrefaciens* and *V*. *parahaemolyticus* and affect flagellar rotation reversals required for cell tumbling and changes in swimming direction [41]. Fold prediction tools detect similarities of the C-terminal VC1909 region to MIT (microtubule interacting and trafficking) and TPR (tetratricopeptide repeat) helical motifs that are often found in scaffolding proteins and can directly mediate protein-protein interactions in multi-component macrocomplexes [42]. Indeed, deletion of the cytoplasmic region of VC1909 resulted in diffuse localization of the GFP fusion, suggesting that interactions through the cytoplasmic domain are indeed essential for the protein’s polar localization (S3I Fig).

### VC2232

Finally, VC2232 is also annotated as a hypothetical protein with unknown function. It is predicted to be synthesized as a signal peptide-containing precursor which is exported in the periplasm and remains anchored to the inner membrane via a single C-proximal transmembrane helix and a short, positively charged cytosolic tail. Following cleavage of the signal peptide, the large periplasmic region is predicted to fold into three immunoglobulin-like domains. The latter are highly stable structural modules that are often found in multiple repeats and can mediate protein-protein interactions in periplasmic or cell surface molecules such as pili, non-fimbrial adhesins, periplasmic chaperones, usher proteins, ABC transporters, and a variety of enzymes [43] (S3A and S3H Fig). Truncated VC2232 mutants show that while the cytosolic tail is dispensable for the protein’s polar localization, its transmembrane region remains essential for proper cell pole targeting (S3J and S3K Fig). Indeed, the membrane-proximal periplasmic amino acids together with the inner membrane anchor are predicted to fold into a continuous coiled coil which likely stabilizes the functional quaternary structure of the protein.

### VC1909 and VC2232 as motility regulators

**Fig 3:**
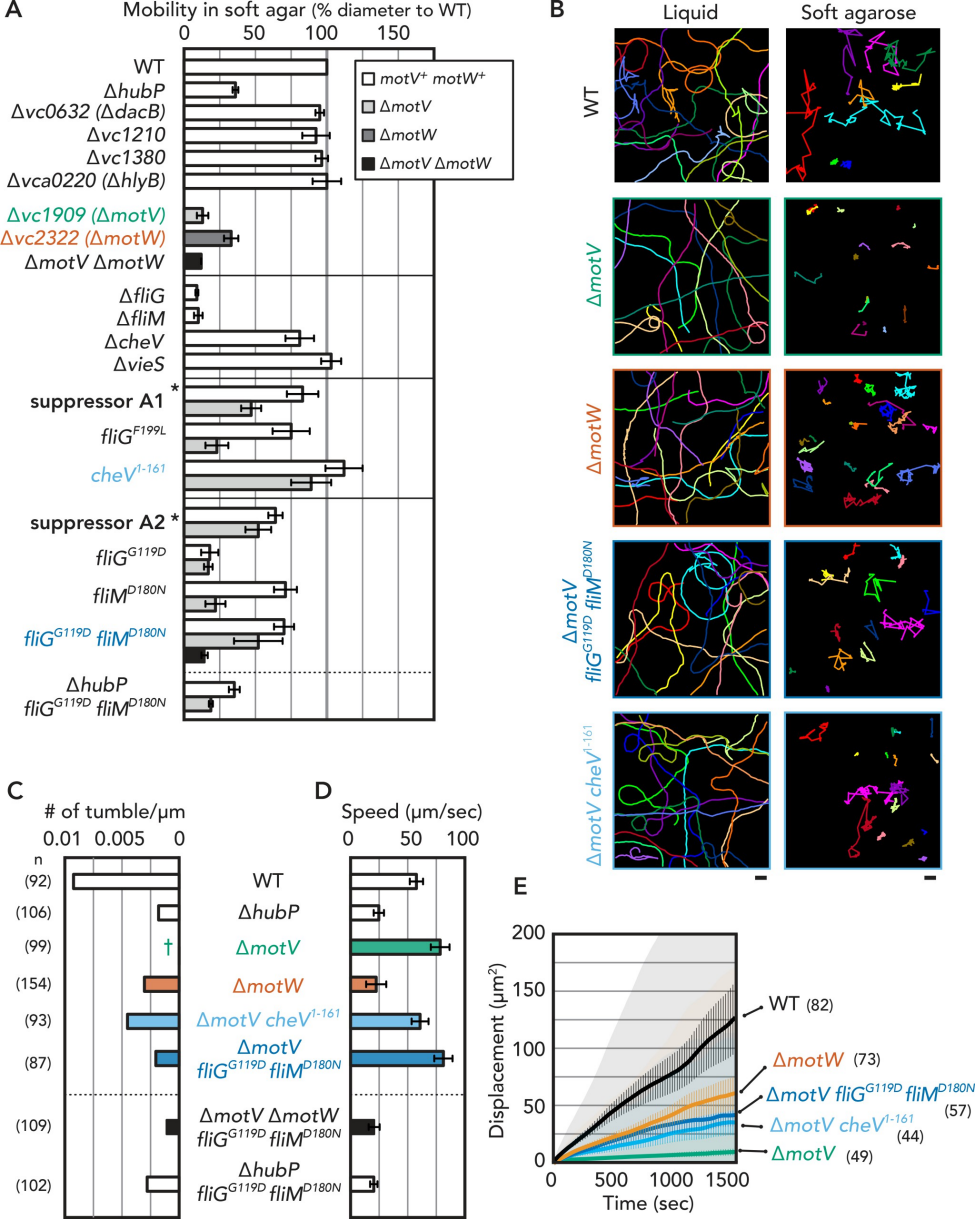
Motility of *V*. *cholerae* mutants. (A) Motility defects examined by swimming in soft agar plates. Average diameter relative to WT, along with standard deviations from at least three experiments are shown. Only limited number of strains are shown and the exhaustive results can be found in S5 Fig. * *motV*^*+*^ complemented in the suppressor strain. (B) Representative trajectories of *V*. *cholerae* cells in liquid (left) and 0.25% agarose (right) environments. Bars = 10 μm. Tumbling frequency (C) and velocity (D) of *V*. *cholerae* cells from video-tracking in liquid. Numbers of cells analyzed (n) are shown on the left in parenthesis. † denotes no tumbling. Standard deviations from at least three replicates are shown for (D). (E) Mean square displacement (MSD) of *V*. *cholerae* cells in 0.25% agarose. Average (thick lines), standard error of the mean (vertical bars), and standard deviations (shades) of projected individual trajectories are shown. n is indicated in parentheses.

Since Δ*hubP* mutants in *V*. *cholerae*, as well as deletion mutants of the *vc1909* homologs in *S*. *putrefaciens* or *V*. *parahaemolyticus* show defects in motility in soft agar plates [11,41], we examined the role of newly identified HubP partners in flagellar motility. As shown in Fig 3A, Δ*vc0632/dacB*_*vc*_, *Δvc1210*, *Δvc1380* and Δ*vca0220/hlyB* mutants are motile with wild-type (WT) levels of motility in soft agar plates. In contrast, the Δ*vc2232* mutant showed motility defects similar to that of the Δ*hubP* mutant, while the Δ*vc1909* mutant was not able to expand its growth outside the inoculated surface. These data indicate direct involvement of not only VC1909, but also VC2232 in motility regulation and hereafter we refer to the two genes as MotV and MotW, respectively. The rest of the study will mainly focus on these two proteins and their involvement in motility.

Motility defects can have different underlying causes such as lack of flagellum assembly or compromised flagellar integrity, expression of multiple appendages leading to lopho-, peri- or amphitrichous flagellation, or defects in the rotational or directional switching dynamics of the assembled polar appendage. Cell imaging by electron microscopy revealed that Δ*motV*, Δ*motW* and Δ*motV* Δ*motW* mutants feature single, seemingly intact flagella at the cell poles, morphologically undistinguishable from those in WT cells (S2D Fig). This result indicates that while Δ*motV* and Δ*motW* mutants are able to assemble flagellar structures, they are likely compromised in the rotational dynamics or directional switching of their motility organelles.

Δ*hubP* cells were shown to maintain their swimming capabilities but display a significant bias toward straight swimming with turning events observed 3–4 times less frequently and with lower velocity than WT cells (Fig 3C and 3D, [11]). To further examine how flagellar motility was affected in the Δ*motV* and Δ*motW* mutants and whether the motility defects correlate with those of the Δ*hubP* strain, we performed single-cell video-tracking experiments. Similar to the Δ*hubP* cells, the Δ*motV* mutant exhibited defects in turning. Nevertheless, these defects were much more severe in the Δ*motV* mutant, with the majority of cells swimming straight across the imaging field and never tumbling for the duration of the video-tracking experiments (Fig 3B and 3C). In addition, the average speed of swimming was somewhat higher in the Δ*motV* mutant compared to WT (Fig 3D), which can be explained by the lack of tumbling and confirms defects in directional switching but not in rotational dynamics of the flagella.

The soft-agar growth phenotype and video-tracking experiments revealed seemingly conflicting results for the Δ*motV* mutant. Indeed, the cells were found to swim with speeds comparable or superior to WT *V*. *cholerae*, however, the colonies were unable to expand from the inoculated soft-agar area. To reconcile these results, we carried video-tracking experiments on cells embedded in semisolid agarose between the microscope slide and cover slip. In these conditions, mimicking the motility assay in soft agar on plates, the Δ*motV* cells were often found stuck against the agarose matrix and exhibited very little displacement ability compared to WT *V*. *cholerae* (Fig 3B and 3E). Together, these results confirm that both the swimming by flagella rotation and directional switching are necessary for colony expansion in non-liquid media, with cell turning likely required to overcome the physical obstacles presented by the media matrix.

In contrast to the Δ*motV* cells, the Δ*motW* mutant showed defects in both the tumbling frequency and swimming speed, indicating a distinct role in motility regulation (Fig 3B, 3C and 3D). Furthermore, we examined the motility of the Δ*motV* Δ*motW* double mutant. Although the double mutant assembled monotrichous flagella similar to WT *V*. *cholerae*, the strain was totally defective in soft agar expansion, similar to the Δ*motV* single mutant. However, more severe motility defects were observed in video-tracking experiments in liquid: the vast majority (85%) of cells were immotile throughout the entire length of recording (~13 seconds), indicating that MotV and MotW work separately to control flagellar motility.

### Interactions and interdependence among HubP, MotV and MotW

Whereas MotV homologs have been recently shown to interact with HubP in bacterial two hybrid assays [41], there are no reports of HubP-dependent cell pole recruitment of MotW homologues in the literature. Microscopy of truncated mutants suggested that MotW’s transmembrane region and periplasmic domains are required for its HubP-dependent targeting to the pole, whereas its short cytosolic tail appears dispensable for proper localization (S3J and S3K Fig). To corroborate interactions between HubP and MotW, we generated several strains encoding FLAGx3- or HA-tagged HubP, MotW and MotV proteins from their endogenous chromosomal loci. Strains expressing FLAGx3 or HA-tagged HubP or MotW exhibited unperturbed morphology, cell growth and motility phenotypes (S4B Fig) and western blot analyses of the cell lysates exhibited specific signals for the respective epitope tags on the two proteins: the SDS-PAGE profile of tagged MotW is consistent with its theoretical monomeric molecular weight in the ~50 kDa range, whereas HubP’s running behavior is consistent with that of a detergent-resistant dimer in the high molecular weight region (S4C Fig). Eluted samples following stringent affinity pull-downs using anti-FLAG or anti-HA affinity resins were found to indeed contain both proteins further indicating direct or indirect interaction between the two polar markers (S4D Fig). Finally, bacterial two-hybrid assays based on the functional complementation between adenylate cyclase fragments fused to HubP’s and MotV’s periplasmic domains further support interaction between the two proteins, which is likely stabilized by additional factors in their native periplasmic context *in vivo*. Similar attempts to detect MotV-HubP interactions were not successful, as the insertion of epitope-encoding sequences at the 5’ or 3’ ends of the *motV* gene invariably led to strains featuring non-motile phenotypes and no MotV-specific bands were detected upon analyses of the corresponding cell lysates (*e*.*g*. S4C Fig).

While MotV-GFP and MotW-GFP exhibited similar bipolar foci in *hubP*^+^ cells when expressed from plasmids (Figs 1C and 2), it is known that protein expression levels can affect uni- and bi-polarity of polar landmark proteins and their interaction partners [12,44]. To avoid possible overexpression effects resulting from the inducible plasmid-based expressions described above, we generated *V*. *cholerae* strains expressing fluorescent fusion proteins at the native *motV* and *motW* loci and under control of the respective endogenous promoters (for expression of MotV-CFP or MotW-CFP, respectively). Although the overall co-localization of the two proteins with HubP remained consistent with the plasmid-based co-localization data, we observed increased proportion of cells carrying unipolar MotW loci in cells showing bipolar HubP (HubP-YFP) fluorescence (Fig 2). In addition, whereas the plasmid-based localization experiments indicated that polar localization of both MotV and MotW is HubP-dependent, the cell pole-targeting of each protein was independent of that of the other (S4A Fig). Together, these results further indicate distinct involvement of MotV and MotW in motility. In addition, different timing for the expression and/or local recruitment of HubP and its downstream interaction partners suggests complex maturation of the cell poles with long-axis asymmetry.

### Suppressor analysis

To further explore the function of MotV and MotW in motility and to understand in which pathways MotV and MotW are involved, we carried out suppressor development. Spontaneous mutants of the Δ*motV* and Δ*motW* strains that showed improved motility could be detected and selected in soft agar plates without treatment with mutagenic agents. (Figs 3A and S5). Whole-genome sequencing of the motility reversal isolates showed that four proteins, VieS (VC1653), FliM (VC2126), FliG (VC2132) and CheV4 (VCA0954), were often found to be mutated in suppressor strains (Table 1). FliG mutations were found in both Δ*motV* and Δ*motW* suppressors, while FliM mutations were only found in Δ*motV* suppressors, together or not with mutations in FliG (Table 1). Interestingly, FliG and FliM interact directly and together with multiple copies of FliN build the flagellar C-ring complex, a conserved cytosolic component responsible for both torque generation and directional switching and therefore essential for flagellar motility. In accordance with this, Δ*fliG* and Δ*fliM* mutants are fully deficient in motility in soft agar plates (Fig 3A).

**Table 1:** Summary of generated strains with suppressor mutations*.

Parental strain	Suppressor	VieS	FliM	FliG	CheV4	Other mutations
Δ*motV*	**A1**	—	—	F199L	1–161	—
	**A2**	—	D180N	G119D	—	VC0079^R69S^, VC2749^V8Δ^
	**A3**	—	G111E	—	E169K	—
	**A4**	—	—	—	—	VC0328^G604A^, VCA0738^A156E^
	**A6**	—	R107C	A59S	—	VC2063^D442N^, *Δvc1152-1153*
	**A8**	—	—	K281E	T30A	—
*ΔmotW*	**B1**	T1030I	—	—	—	VC0292^V20A^
	**B2**	—	—	V54M	—	VC2453^H870Y^, VC0290^R85H^
	**B4**	H1002Y	—	—	—	*vc0291*::*IS1004*
	**B6**	A669V	—	—	—	*IG_vca0695-7* ^†^
	**B7**	—	—	G119D	—	VC0377^P8L^
	**B8**	—	—	—	—	VCA1031^A391V^

*—, not applicable.

^†^ T to A nucleotide substitution at position 634,840, located in the intergenic region between *vca0695* and *vca0697*.

### MotV suppressors

**Fig 4:**
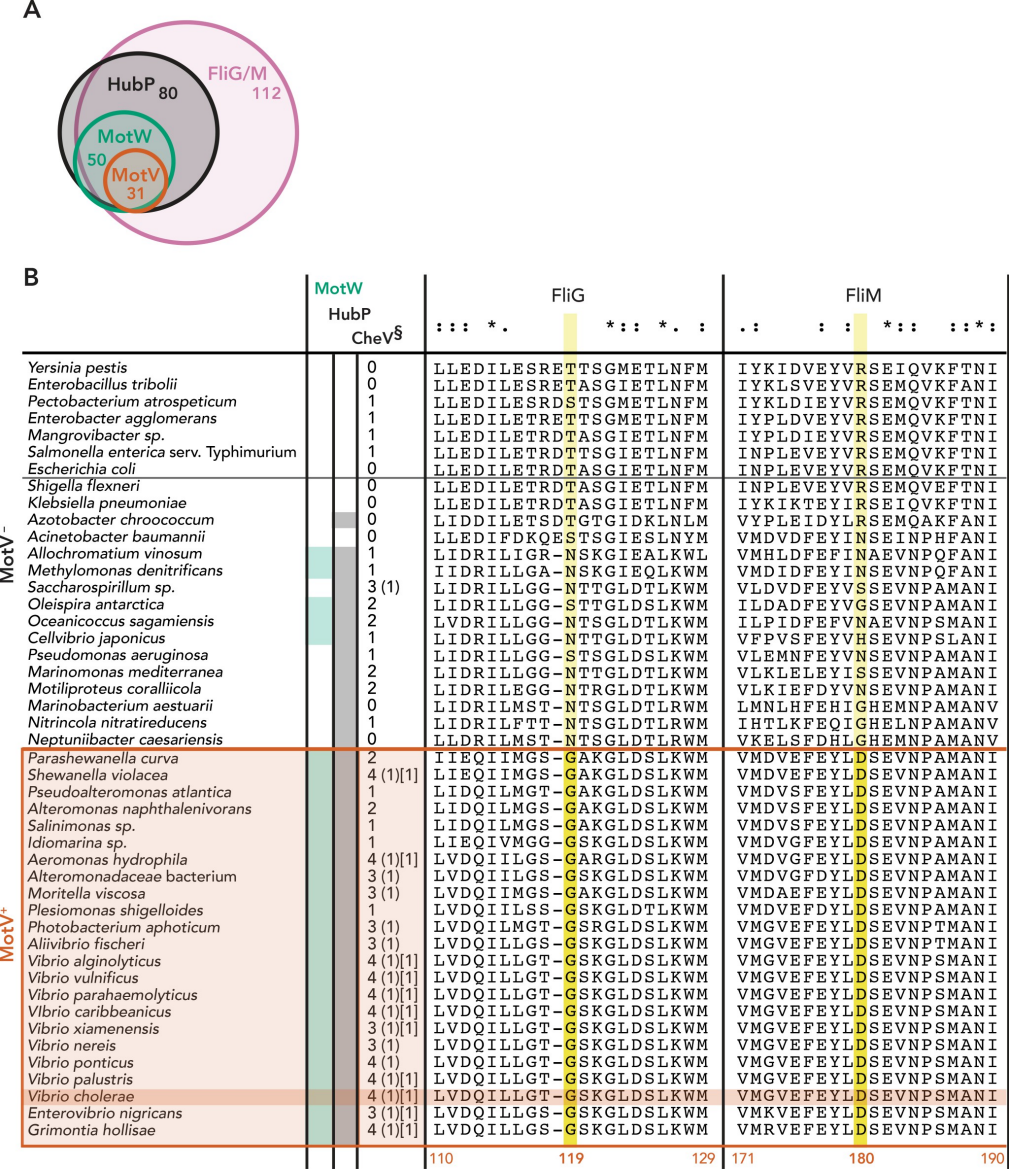
Multiple sequence alignments of FliG and FliM. (A) Presence of C-ring (FliG/M), HubP, MotV and MotW in 132 γ-proteobacteria species shown in Venn diagram. (B) Alignments of FliG and FliM from different γ-proteobacterial species. For each organism, presence of HubP, MotW and CheV homologs are indicated. (§) In addition to total number of CheV proteins, number of CheV variants with non-canonical ‘switch’ residues are indicated in round (A substitution) and square [T substitution] brackets (see text and Fig 5). Asterisk (*), colon (:), and period (.) indicate amino acids with full conservation, strongly similar properties, and weakly similar properties, respectively. Key residues differentially conserved in MotV^+^ versus MotV^-^ bacteria are labeled in yellow.

**Fig 5:**
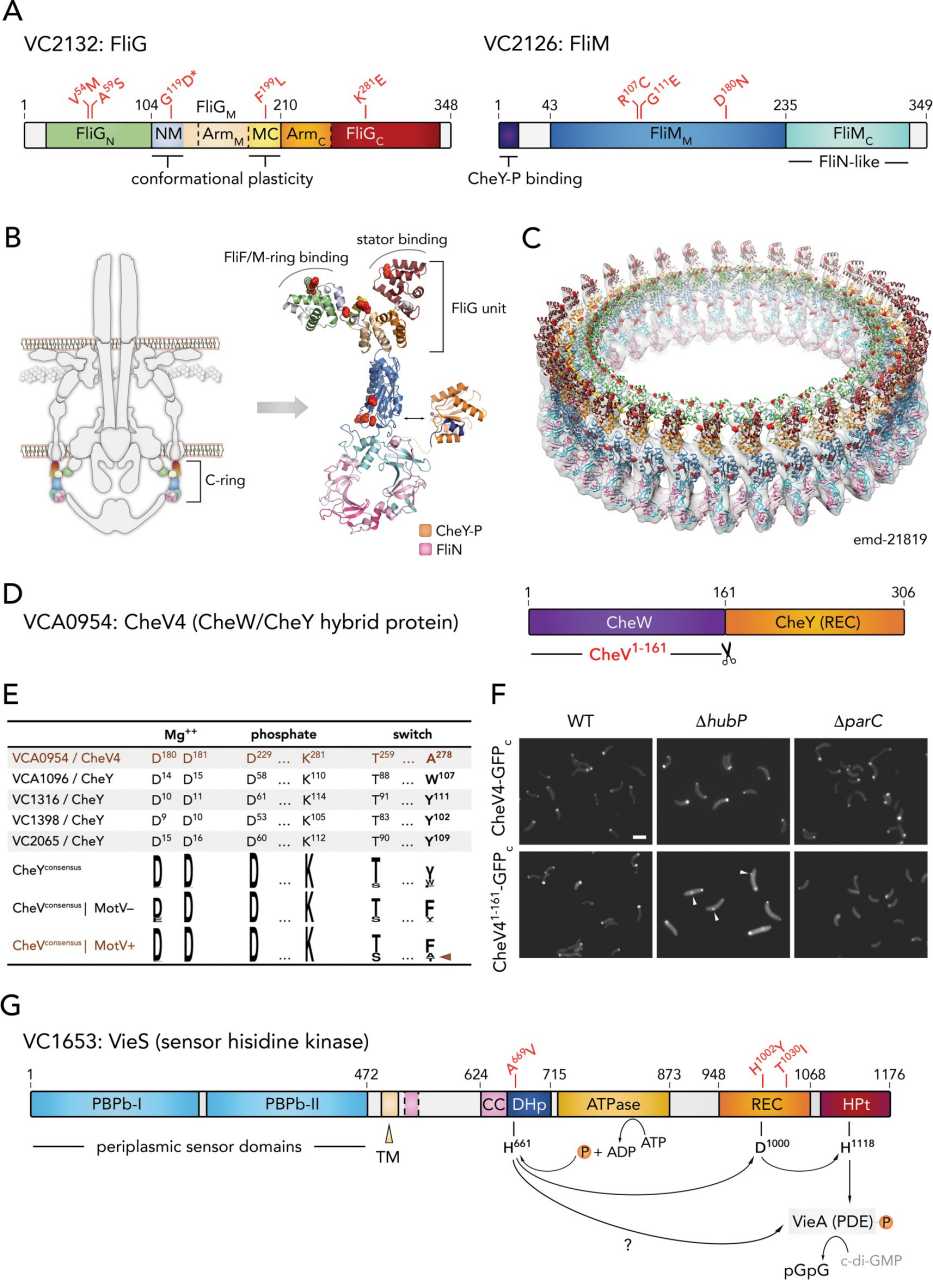
Motility regulators with identified Δ*motV* and/or Δ*motW* suppressor mutations. (A) Domain architecture of conserved C-ring component FliG (Left) and FliM (right). _N_, N-terminal domain; _M_, middle domain; _C_, C-terminal domain; NM, FliG_N_-FliG_M_ domain linker; Arm, armadillo repeat-like motifs; MC, FliG_M_-FliG_C_ domain linker; CheY-P, phosphorylated CheY. (B) Left, schematic of the *V*. *cholerae* flagellum with C-ring components in color. Right, cartoon representation of a homology model of a single FliG-FliM-FliN-CheY unit with color coding as in (A) and based on structural information from [45]. FliG and FliM amino acids mutated in the suppressor strains are highlighted as red spheres. The thumbnail representation of the flagellum is adapted with modifications from [45], under the license CC BY 4.0 (https://creativecommons.org/licenses/by/4.0/legalcode). (C) A three-dimensional homology model of the assembled multi-copy FliG-FliM-FliN C-ring with indicated positions (red spheres) of the suppressor mutations are as in (B). (D) VCA0954/CheV4 domain architecture. REC, receiver domain. (E) Key residues involved in receiver domain phosphorylation as compared to canonical CheY proteins and CheV homologs in MotV^−^and MotV^+^ species. (F) Representative fluorescence microscopy images of cells expressing full length (top rows) or truncated (bottom rows) CheV4*-GFP fusions from the native *cheV* chromosomal locus in different genetic backgrounds, as indicated. Non-polar fluorescence foci are indicated with white arrowheads. Bar = 2 μm; (G) Predicted VC1653/VieS domain architecture. PBPb, bacterial periplasmic substrate-binding protein domain; TM, transmembrane region; CC, coiled-coil; DHp, dimerization histidine phosphotransfer domain; ATPase, ATP binding and hydrolysis domain; HPt, histidine phosphotransfer domain; PDE, phosphodiesterase; c-di-GMP, cyclic diguanylate; pGpG, linear diguanylate.

As opposed to the ubiquitous C-ring components, only a limited subset of flagellated bacteria encode MotV and MotW homologs (Fig 4A). Given the emergence of multiple suppressor strains with mutations in the *fliG* and *fliM* genes, we hypothesized that *motV*^+^/*motW*^+^ bacteria could possess distinct amino acid residues or motifs in their FliG and/or FliM components that are responsible for MotV- and/or MotW-specific regulatory inputs through the C-ring. Indeed, phylogenetic analyses of FliG and FliM identified amino acids conserved only in *motV*^+^/*motW*^+^ species. Remarkably, FliG^G119^ and FliM^D180^ were among these residues and both were found to be mutated in the Δ*motV* suppressor mutant A2 (Table 1 and Fig 4B). FliG^G119^ maps at the linker connecting the N-terminal and middle domains of the FliG C-ring unit, which has been proposed to undergo significant conformational changes during directional switching of the flagellum. FliM^D180^, on the other hand, maps on the inner surface of FliM’s middle domain, close to the interface with the protein’s C-terminal domain (Fig 5A, 5B and 5C). Importantly, the two residues are physically distant and do not partake in pairwise interactions (Fig 5B and 5C).

To further examine the functional significance of these co-evolved residues and their role in Δ*motV* suppression, we tested the effects of FliG^G119D^ and FliM^D180N^ mutations by introducing them into WT and Δ*motV* background, independently or in combination. In the *motV*^+^ background, the *fliG*^G119D^ mutation abolished the motility in soft agar, similar to *fliG* deletion, indicating functional incompatibility. In contrast, the *fliM*^D180N^ *motV*^+^ mutant retained ~80% of motility relative to WT (Fig 3A), but introducing the same *fliM*^D180N^ mutation in a Δ*motV* background did not rescue the immotile phenotype. Strikingly, introducing the *fliM*^D180N^ mutation in the *fliG*^G119D^ *motV*^+^ background overrode the motility defect of the *fliG*^G119D^ mutation (Fig 3A). Furthermore, a *fliG*^G119D^ *fliM*^D180N^ Δ*motV* triple mutant retained motility in soft agar, at ~50% relative to WT *V*. *cholerae* and comparable to that of suppressor mutant A2, which carries some additional mutations (Table 1 and Fig 3A). In addition to motility in soft agar, video-tracking experiments indicated that the *fliG*^G119D^ *fliM*^D180N^ Δ*motV* triple mutant recovered the ability to tumble (Fig 3B). Furthermore, this mutant swam as fast as the Δ*motV* mutant in liquid and was not trapped in the 0.25% agarose matrix (Fig 3B, 3D and 3E). In contrast, FliG^G119D^ and FliM^D180N^ mutations did not suppress motility defects by Δ*hubP* or Δ*motW* (Fig 3A). Together, these results suggest that the combination of FliG^G119D^ and FliM^D180N^ mutations were sufficient to suppress the effect of Δ*motV*, probably transforming the C-ring to be function-independent of the MotV protein.

Δ*motV* suppressor A1 was also found to harbor a mutation in *fliG*, FliG^F199L^, which maps at the second FliG interdomain linker (MC) known to undergo significant conformational changes upon directional switching (Fig 5A). Introducing the FliG^F199L^ mutation in Δ*motV* background slightly enhanced motility in soft agar but the rescue phenotype was not comparable to that of suppressor A1. Interestingly, A1 also features a 14-base-pair deletion in *cheV4*, leading to a frameshift from the protein’s 161th amino acid and emergence of a stop codon 2 amino acids later (thus the mutant will be referred here as CheV4^1-161^). Remarkably, introducing *cheV4*^1-161^ to the Δ*motV* mutant was sufficient to rescue motility in soft agar and the resulting *ΔmotV cheV4*^1-161^ cells also showed restored ability to swim and tumble in video-tracking analyses (Fig 3B and 3C). CheV proteins are CheW-CheY hybrids (Fig 5D), where the CheW module is proposed to bridge a CheA histidine kinase to its cognate MCP and allow phosphorylation of the CheY module, which in turn would bind the N-terminal region and middle domain of FliM to favor flagellar directional switching and cell tumbling. The observed suppressor mutation *cheV4*^*1-161*^ is equivalent to removal of the CheY module from the CheV4 protein, possibly by allowing the CheW module to transmit sensory inputs in a MotV-independent manner through interactions with canonical CheY proteins. CheV4 and CheV4^1-161^ localize at the cell pole (Fig 5F) and feature mainly unipolar localization similar to that of the better-studied CheW1 protein [22,23]. Unlike CheW1, however, CheV4 and CheV4^1-161^ remained polar and without additional foci in a Δ*parC* background. Interestingly, CheV4 was found to be enriched in the HubP^+^ minicells (S1 Table). Indeed, in a Δ*hubP* background CheV4 distribution shows the emergence of additional foci, suggesting that polar localization of the CheV4 protein could be at least partly HubP- and/or MotV-dependent (Fig 5F).

### MotW suppressors

VieS mutations were only found in Δ*motW* suppressors and not in Δ*motV* suppressors. VieS is a hybrid sensor kinase which has been shown to positively regulate motility by activating the phosphodiesterase (PDE) VieA and altering the cellular levels of the second messenger c-di-GMP [46]. Interestingly, observed VieS suppressor mutations (VieS^A669V^, VieS^H1002Y^ and VieS^T1030I^) map at or near residues involved in canonical phosphotransfer through the protein’s intrinsic receiver domain, raising the possibility for direct phosphorylation of the cognate phosphodiesterase instead (Fig 5G). Although а Δ*vieS* mutant did not show motility defects in our hands, VieS mutants with any of the above single amino acid substitutions exhibited hypermotile phenotypes in soft agar plates (S5 Fig), suggesting that motility defects of the Δ*motW* mutant could be compensated by VieS/VieA-induced hypermotility in the suppressors.

## Discussion

**Fig 6:**
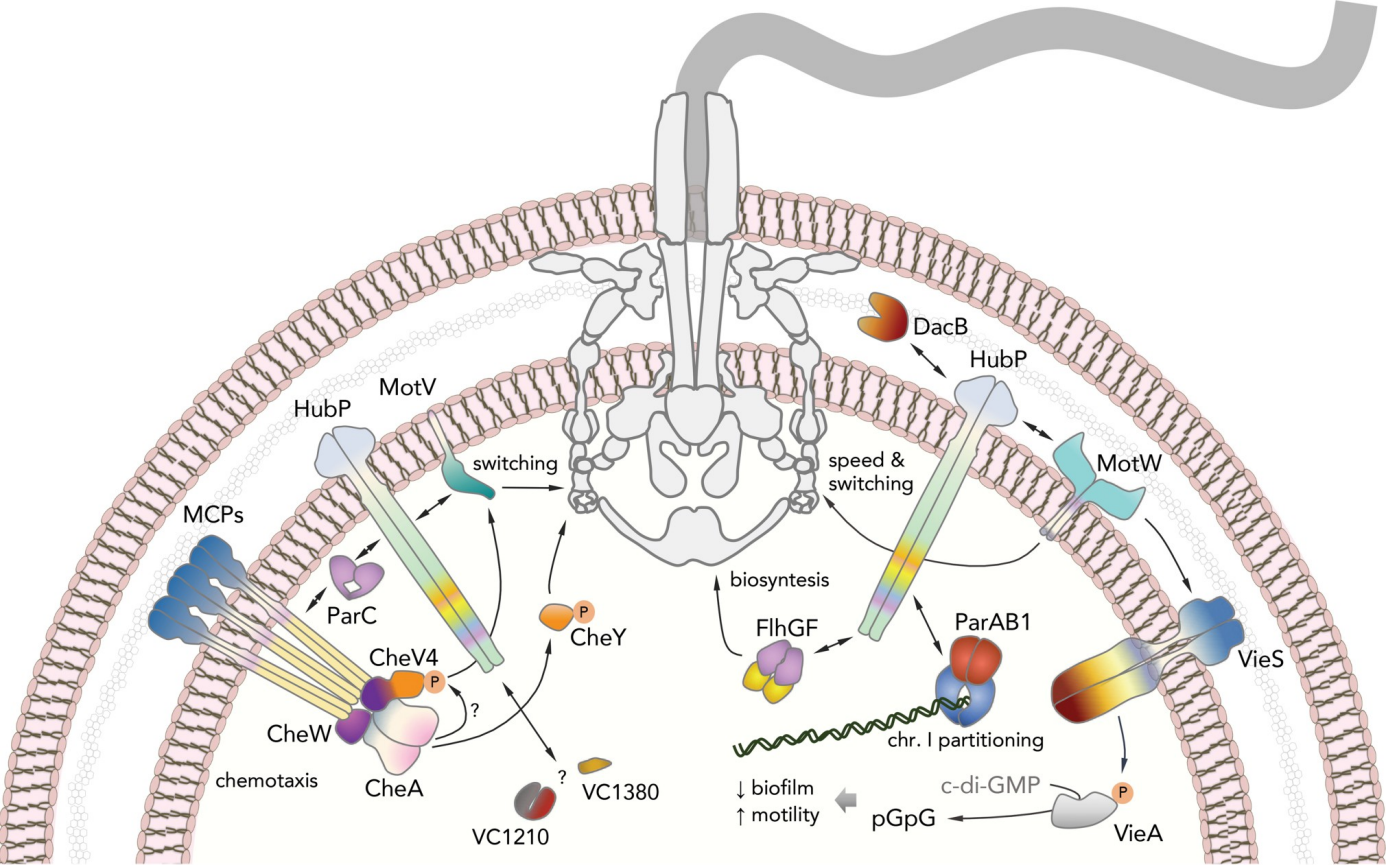
HubP-dependent cell pole organization. Proposed integrated model for HubP-dependent cell pole organization and functional linkages among HubP, identified partners, flagellar motility, chemotaxis signaling and c-di-GMP signaling pathways. The thumbnail representation of the flagellum is adapted with modifications from [45], under the license CC BY 4.0 (https://creativecommons.org/licenses/by/4.0/legalcode).

Although first described several decades ago, engineered bacterial minicells have recently gained popularity as a tool to study a variety of cellular processes and subcellular structures. Examples include but are not limited to the *in situ* three-dimensional visualization of large molecular complexes by cryo-electron tomography [47], characterization of the spatiotemporal mRNA organization by comparative transcriptomics [48] or identification of asymmetric membrane protein distribution by proteomic approaches [26]. In this study, we took advantage of cell pole-derived minicells to identify proteins specifically recruited to the *V*. *cholerae* cell pole by the polar landmark protein HubP. Comparative proteomics analyses followed by fluorescence microscopy-based imaging of pole-targeted candidates led to the identification of four novel HubP partners with likely roles in cell wall remodeling, chemotaxis and flagellar motility (DacB, HlyB, MotV and MotW), as well as two more uncharacterized cytosolic proteins (VC1380 and VC1210). An integrated model of HubP-dependent cell protein targeting and here-in described functional linkages is shown in Fig 6.

DacB is also known as PBP4, a periplasmic low-molecular weight penicillin-binding protein with peptidoglycan DD-endopeptidase and DD-carboxypeptidase activities. Although the protein is not essential for growth in *E*. *coli* or *V*. *cholerae*, its specific targeting to the bacterial pole might contribute to asymmetric cell wall remodeling during cell growth or proliferation. For example, overexpression of DacB homologs can lead to cell lysis in *E*. *coli* and has been shown to induce aberrant cell shape and loss of polarity in certain developmental transitions in *Vibrio parahaemolyticus* [49,50]. In addition, DacB_Ec_ has been proposed to collaborate with the major peptidoglycan amidases during daughter cell separation as DacB_Ec_ deletion in strains lacking AmiC leads to the formation of long chains of cells concatenated at the poles [51]. Importantly, we previously showed that HubP’s own periplasmic peptidoglycan-binding LysM domain is both sufficient and necessary for the protein’s targeting to the pole [11]. Taken together, these observations indicate that albeit not essential, the HubP-dependent positioning of DacB_Vc_ at the *Vibrio* cell poles could facilitate cell shape maintenance and secure cell pole-specific peptidoglycan remodeling for correct ultrastructural organization, cell growth, and proliferation (Fig 6).

For two other of the here-in identified HubP partners, MotV (VC1909) and MotW (VC2232), we showed that their deletion mutants exhibited severe growth defects when inoculated onto soft agar plates. Radial growth on soft agar has been widely used as a measure of bacterial chemotactic motility. For example, disruptions of genes encoding chemotaxis signaling proteins or known regulatory, structural, motor or type III secretion system proteins of the flagellum have been shown to induce similar robust defects in soft agar motility [40,52]. In addition, forward genetic screens of motility mutants using transposon insertion libraries have previously helped unveil novel motility regulators. These include not only proteins directly involved in flagellar assembly, such as the *C*. *crescentus* TipF and *V*. *cholerae* FlgT proteins [31,53], but also some of the landmark cell pole organizers of various species, including *V*. *cholerae*’s HubP itself [11,53]. Interestingly, while MotV homologs in *A*. *fischeri*, *V*. *parahaemolyticus* and *S*. *putrefaciens* were recently identified as motility regulators in forward genetic screens for identification of motility-deficient mutants [40,41], there are no reports on MotW or its role in flagellar motility to date.

Consistent with studies on MotV homologs [40,41], the Δ*motV V*. *cholerae* mutant showed severe defects in soft agar motility and in-liquid tumbling frequency, whereas the overall swimming velocity in liquid remained similar to that of WT cells. Our video-tracking experiments in semi-solid environment further demonstrate that the lack of cell tumbling leads to bacterial immobilization onto the media matrix despite the seemingly intact flagellar torque generation. Interestingly, both studies on the *S*. *putrefaciens* MotV homolog ZomB [41] and the here-in presented data on Δ*motV* suppressor development suggest a strong genetic interaction between MotV and flagellar C-ring components FliM and FliG. However, whereas a deletion of the C-terminal 14 residues in *S*. *putrefaciens* FliM proved sufficient to partially rescue motility defects of a *zomB* (*motV*_*Sp*_) deletion in the bacterium [41], it is important to note that this region is not conserved among the otherwise highly conserved FliM homologs. In contrast, we identify here suppressor mutations in both FliG and FliM at non-interacting residues whose conservation appears to strongly correlate not only between them but also with the presence of a *motV* homolog in the genome (FliG^G119^ and FliM^D180^) (Table 1 and Fig 4B). Together with multiple secondary FliG and FliM mutations observed in the various Δ*motV* suppressors identified here (Table 1 and Fig 5A, 5B and 5C), these data strongly suggest functional coevolution of the C-ring components with their cognate MotV homologs to provide additional regulatory inputs for flagellar motor reversals (Fig 6).

In addition to FliG and FliM, our Δ*motV* suppressor analyses also identified specific mutations in VCA0954, which has been annotated as *V*. *cholerae* CheV4. As mentioned above, CheV proteins are CheW-CheY hybrids and the suppressor mutation is equivalent to deletion of the entire C-terminal receiver domain. Interestingly, while stand-alone deletion of the CheV4^CheY^ module (CheV4^1-161^) is sufficient to complement the motility defects in Δ*motV* background, deletion of the entire *cheV4* gene failed to rescue the motility phenotype (S5 Fig). Furthermore, both full-length CheV4 and CheV4^1-161^ localized at the cell pole, even in Δ*parC* background (Fig 5F), and suppressor mutations were not detected in any of the remaining *cheV* or *cheY* genes (Table 1). A closer analysis of the CheV4^CheY^ domain reveals a deviation from the canonical CheY consensus at key amino acid positions involved in phosphorylation. Although not universal, CheY activation typically involves the so-called ‘switch’ residue pair–two highly conserved amino acids with hydroxyl (Ser/Thr) and aromatic (Tyr/Phe/Trp) side chains, corresponding to residues 259 and 278 in CheV4, respectively (Fig 5E). Upon classical CheY domain phosphorylation, the hydroxyl ‘switch’ residue would reorient to form a hydrogen bond with the active site phosphoaspartate (CheV4^D229^), whereas the aromatic side chain would flip to occupy the cavity vacated by its ‘switch’ partner [54]. CheY phosphorylation would increase dramatically the protein’s affinity for binding to the FliM N-terminus at the C-ring leading to flagellar motor reversal and cell tumbling. In CheV4, however, the aromatic residue position is occupied by an alanine instead of a bulky aromatic amino acid, suggesting that the protein might be intrinsically compromised in its phosphorylation efficiency. Gene co-occurrence analyses show that, similar to *V*. *cholerae*, *motV*^*+*^ bacteria generally encode multiple CheV proteins, one or more of which harbor an alanine or threonine substitution at the aromatic ‘switch’ residue position (Figs 4 and 5). Together, these data strongly suggest a direct role of CheV4, in addition to the C-ring FliM and FliG components, in MotV-dependent motility control (Fig 6). Similar to the C-ring suppressor mutants, removal of the CheV4^CheY^ domain in the A1 suppressor could lead to CheV4^CheW^ coupling with canonical CheY proteins to preserve the signaling input while rendering the protein MotV-independent.

While some bacterial polar landmark proteins such as DivIVA and PopZ are cytoplasmic proteins, HubP is an inner membrane protein featuring both periplasmic and cytosolic modules. Nevertheless, previously characterized interaction partners are either cytosolic proteins [11,15,55,56] or inner-membrane proteins interacting with HubP’s large cytosolic domain [13]. In contrast, we here identified DacB, a periplasmic protein, and MotW, an inner membrane-anchored protein with large periplasmic domains, as novel HubP-dependent polar proteins. Protein localization analyses on truncation MotW variants indeed indicated that the protein’s periplasmic domains, and not its C-terminal cytosolic tail, are essential for the HubP-dependent targeting to the pole (S3J and S3K Fig), whereas protein pulldown experiments and bacterial two-hybrid complementation assays support a physical interaction between HubP and MotW, which could be strengthened by additional binding partners *in vivo* (S4D and S4E Fig). It is thus intriguing to see that HubP acts as a *V*. *cholerae* master pole organizer that arranges not only the cytoplasmic space but also the periplasmic compartment (Fig 6).

Gene co-occurrence analyses showed that while there are many species that encode HubP but not MotV homologs, *motV* genes are found exclusively in *hubP*-positive species (Fig 4). Similarly, *motW* homologs were found almost exclusively in bacteria harboring *hubP*-homologous genes, however, the presence of *motW* genes did not necessarily correlate with that of *motV*. In addition to their independent genetic organization, the subcellular localization of the encoded MotV and MotW proteins also appeared unaffected by the presence or absence of the other HubP partner (S4A Fig). Finally, our soft agar motility assays and single-cell video-tracking experiments also demonstrated different motility defects between the Δ*motV* and Δ*motW* mutants: defects in soft agar motility in the Δ*motW* mutant were milder than those in the Δ*motV* background and video-tracking experiments demonstrated that the *motW* deletion leads to both reduction in tumbling frequency and swimming velocity in liquid, whereas lack of *motV* affected only cell tumbling but not swimming velocity. This, together with the independent protein localization patterns and genetic distribution of the two HubP partners supports a model for distinct mechanisms of motility regulation by the two proteins. It is therefore surprising that one of the Δ*motW* suppressors, B7 (Table 1), features the same the FliG^G119D^ substitution identified in Δ*motV* suppressor A2, that would normally render the cells immotile in WT *motV* and *fliM* backgrounds (Figs 3A and S5). Interestingly, the B7 suppressor also features a secondary mutation in *vc0377*, which encodes a protein belonging to the CheX family of protein phosphatases that are proposed to regulate motility by CheY dephosphorylation [57]. Although the role of VC0377 in *V*. *cholerae* remains to be experimentally determined, these results paint an ever more complex picture of multiple converging inputs for motility regulation.

Another set of Δ*motW* suppressor mutations clustered in the *vc1653* gene encoding the VieS hybrid sensor kinase, which is involved in c-di-GMP signal transduction through specific activation the VieA phosphodiesterase (PDE) (Table 1 and Fig 5G) [46]. In addition, suppressor B6 also features a secondary mutation in the intergenic region preceding *vca0697*, which encodes a GGDEF domain-containing protein with preserved active and I-site motifs (G^560^GEEF and R^551^ESD, respectively) and therefore likely to partake in c-di-GMP signaling as an active diguanylate cyclase (DGC). Cell motility and biofilm formation are typically antagonistic processes of adaptation and the intracellular second messenger c-di-GMP plays critical roles to inhibit the former and induce the latter in response to specific environmental signals [58]. PDE activation and/or DGC inhibition can thus lead to lowering of the cellular c-di-GMP concentrations and stimulate flagellar motility (Fig 6). Whereas previous reports have observed reduced motility in a Δ*vieS* mutant [46], we did not observe significant effects upon *vieS* deletion. Nevertheless, all three point mutations identified in the Δ*motW* suppressors led to hypermotile phenotypes in WT background consistent with VieA activation and c-di-GMP degradation. Interestingly, the VieS mutations map either at the protein’s dimerization and histidine transfer (DHp) domain (VieS^A669V^) or at or near residues that would participate in phosphorelay through the protein’s intrinsic receiver domain (VieS^H1002Y^ near the phosphoaccepting D1000 or VieS^T1030I^ at the canonical ‘switch’ hydroxyl residue (see above)) [59]. These observations raise the possibility of facilitated phosphotransfer directly onto the VieA protein for increased phosphodiesterase activity and stimulated flagellar motility (Fig 6). Whether VieS contributes directly to MotW-dependent motility regulation or the Δ*motW* defects are compensated indirectly by c-di-GMP decrease-dependent motility stimuli remains to be further examined.

## Materials and methods

### Bacterial strains, plasmids and media

Unless otherwise noted, the whole-genome sequenced N16961 strain [60] was used as WT *V*. *cholerae*. Plasmids were constructed by either conventional digestion-ligation methods or by isothermal assembly [61]. List of bacterial strains, plasmids, and oligonucleotides are indicated in Supporting information S2, S3 and S4 Tables, respectively. All expression constructs were verified by Sanger DNA sequencing (GATC Biotech/Eurofins Genomics, Ebersberg, Germany). Mutants were generated by allelic exchange [62]. Plasmids were introduced in *V*. *cholerae* by electroporation. Unless otherwise specified, bacterial cells were grown in lysogeny broth (LB) or LB agar (1.5%) and, as appropriate, antibiotics were used at the following concentrations: ampicillin 100 μg/mL, chloramphenicol 25 μg/mL (for *E*. *coli*) or 5 μg/mL (for *V*. *cholerae*), kanamycin 25 μg/mL, streptomycin 100 μg/mL. Cell growth curves and growth rate measurements were done by microplate reader (Tecan Infinite M200 Pro, Tecan, Switzerland) [63].

### Bacterial two-hybrid assay

In order to avoid false positives resulting from spontaneous reversal of the BTH101 *cyaA* mutant strain [64], the bEYY2122 host strain was first from constructed from the latter by deletion of the adenylate cyclase *cyaA* gene altogether via transduction of *ΔcyaA*::*kan* followed by flipping out of the *kan* resistance cassette. Two test plasmids encoding complementary adenylate cyclase fragments fused to the protein domains of interest, the Gcn4 leucine zipper motif (positive control) or without additional protein sequences (negative control) were subsequently introduced and liquid cultures of cotransformant colonies were spotted on M9 maltose agar plate containing 50 μg/mL of 5-Bromo-4-Chloro-3-Indolyl β-D-Galactopyranoside (X-gal), 500 μM of Isopropyl β-D-1-thiogalactopyranoside (IPTG) and appropriate antibiotics. The plates were then incubated for 2 days at 30°C and protein interactions were evaluated by both colony growth and development of blue coloration resulting from functional complementation of the split adenylate cyclase protein.

### Minicells preparation

Overnight cultures of bEYY1208 (*ΔminD ΔparA1*) and bEYY1319 (*ΔminD ΔparA1 ΔhubP*) were prepared in LB broth at 37°C with agitation (170 rpm). 3 mL of bEYY1208 and 7 mL of bEYY1319 overnight culture were inoculated in 3 L and 7 L of fresh LB media, respectively, and the cell cultures were grown until OD_600 nm_ = 0.8. Cells were harvested as follows: rod cells were sedimented by low speed centrifugation (3000 *g* at 4°C) for 7 min, then the supernatant was transferred into a new flask and centrifuged at higher speed (9000 g at 4°C) for 20 min. The pellet, enriched in minicells, was resuspended in LB and the series of centrifugations was repeated with an extended low-speed step of 40 min. Purity of the minicell preparations was examined by microscopy and viability on LB plate. Total protein amounts in the minicell preparations were quantified by Coomassie (Bradford) Protein Assay Kit (Pierce Biotechnology, Rockford, IL). Purified pelleted minicells with > 11 μg of total protein content were flash-frozen in liquid nitrogen. Two replicates of each mutant (*hubP+* and *hubP-*) were subjected to iTRAQ by ITSI-BIOSCIENCES (Johnstown, PA).

### Fluorescence microscopy

Cells were grown in M9 minimal media supplemented with glucose (0.2%), casamino acids (0.1%) and thiamine (1 μg/mL) at 37°C with agitation (180 rpm). When necessary, fluorescent protein fusions encoded on overexpression plasmids were induced with 0.02% arabinose for 1 h prior to analysis. Cells in exponential phase were spotted on agarose pads (1% agarose in 1x M9 media) that was mounted on the glass slide. Cell images were acquired using DM6000-B (Leica, Wetzlar, Germany), Zeiss Observer.Z1 (Carl Zeiss, Oberkochem, Germany) and Eclipse Ti (Nikon, Tokyo, Japan) microscopes with the Metamorph software (Molecular Devices, San Jose, CA). Image J, Adobe Photoshop and an ImageJ plug-in MicrobeJ [65] were used for image processing and analyses.

### Motility assays

For the soft agar plate-based assays, cells were grown overnight in LB at 37°C with agitation. 1 μL of culture was pricked into semisolid LB agar (0.3%) plates. The WT strain was always included in each test plate as standard. Plates were incubated overnight and the swimming diameter of each strain was measured and compared to the control.

For video-tracking, cells were grown in EZ Rich Defined Media (Teknova Inc, Hollister, CA) to exponential phase. For liquid environments, 2 μL of each culture were directly spotted onto the microscope slide. For semisolid environments, each cell culture was first mixed with agarose (final concentration 0.25% w/v) in EZ media and then 2 μL of the mixture was placed on a microscope slide. After placing a coverslip on top, images were recorded with the Leica microscope (see above). For liquid environments, we used a streaming acquisition function with 37 msec frequency of image capture. For semisolid environments, images were taken every 20 sec for 30 min. At least two individual experiments with three replicates were performed. Trajectories of individual cells were tracked using the ImageJ plug-in MTrackJ [66]. The swimming speed of each cell was calculated as the total travel distance divided by the total time spent in the viewing field, thus cells with more tumbling could be assigned smaller swimming speed values. The number of tumbling events was counted, and the tumbling frequency was calculated as the sum of the travel distance divided by the total number of tumbling events from each sample. For the semisolid environments, we implemented mean square displacement (MSD) analysis using a Matlab script as described previously [67].

### Electron microscopy

2-mL WT and mutant *V*. *cholerae* cultures were grown to exponential phase in antibiotics-free LB medium and under mild agitation to prevent surface shearing of the flagella. Cells were gently sedimented, washed with 1x Tris-buffered saline (TBS) and resuspended in the same buffer. 5 μL of the cell suspension were spotted on glow-discharged carbon-coated copper grids (Agar Scientific, Stansted, United Kingdom). After 1 min incubation, the extra liquid was blotted off and the grids were passed sequentially through three drops of 2% w/v uranyl acetate solution, with 15–30 sec incubation in the last drop, before blotting and air-drying. Images were taken on a 200 kV Tecnai F20 Thermo Fischer transmission electron microscope equipped with an Eagle 4k x 4k CCD camera at nominal magnification of 5000 x for the representative micrographs in S2D Fig. and a range of lower magnifications to allow throughput for flagella vs. cell number quantification.

### Suppressor analysis

Spontaneous suppressor mutants of Δ*motV* and Δ*motW* strains that show restored motility were isolated as follows. 1 μL (~10^6^ cells) of overnight culture of the test strain was inoculated in the motility plate and incubated for overnight at 37°C. Cells that traveled farthest were recovered for the second round of screening. After 4 (for Δ*motV*) or 5 (for Δ*motW*) rounds of screening in the motility plate, single colonies were isolated and confirmed for the suppressor phenotype. For each Δ*motV* and Δ*motW* strain, 8 independent experiments were carried out and 6 mutants were arbitrarily selected for whole-genome sequencing (WGS).

5 μg of genomic DNA in 130 μL H_2_O was sheared with a Covaris S220 focused ultrasonicator (Covaris, Wobum, MA) with target peak at 500 bp. After blunting the DNA ends with T4 DNA polymerase (New England Biolabs, Ipswich, MA) and Klenow Enzyme (Roche, Basel, Switzerland) followed by A-tailing with Klenow Fragment (New England Biolabs), adaptor DNA [68] was ligated. DNA fragments ranging 400~900 bp in size were purified using Pippin Prep (SAGE Science, Beverly, MA), then amplified with Illumina primers PE1.0 and PE2.0. PCR products were purified using QIAGEN MinElute columns (QIAGEN, Hilden, Germany) then size-selected using AMPure XP beads (Beckman Coulter, Brea, CA).

Illumina sequencing (Single-end 150 bp or Paired-end 75 bp) results were accordingly trimmed and mapped to the bEYY1013 sequence using Bowtie I [69] or Burrows-Wheeler Aligner [70]. Bam files were converted to mpileup format then SNPs were identified by a script VarScan [71], as well as visual screening with Integrative Genomics Viewer [72]. Final confirmation of SNPs and small deletions were made by PCR followed by Sanger sequencing. WGS data is available from the ArrayExpress database (accession number # E-MTAB-10691).

### Bioinformatics

Prediction of protein topology and modeling of protein folds was carried out with Phobius [73], GenomeNet (https://www.genome.jp/en/release.html), Robetta (https://robetta.bakerlab.org), Phyre2 (http://www.sbg.bio.ic.ac.uk/~phyre2), CCfold (http://pharm.kuleuven.be/Biocrystallography/cc) and AlphaFold [74]. Protein fold visualizations were carried out in Pymol (Schrödinger, New York, NY) and Chimera UCSF (https://www.rbvi.ucsf.edu/chimera). Gene co-occurrence was analyzed with STRING (https://string-db.org/) and phosphorylation residue conservation was visualized with WebLogo (https://weblogo.berkeley.edu). For phylogenetic analyses, we used gammaproteobacterial species available in the UniPlot database (https://www.uniplot.org), excluding ones with very small genome (less than 3000 genes) and redundant species. FliG and FliM homologs were retrieved using their pfam numbers (PF01706 and PF02154, respectively). Alignments were done by ClustalOmega [75] followed by visualization in Unipro UGENE [76].

### Pull-down assays

For epitope tagged protein expression and identification of HubP- and MotW-specific bands, *V*. *cholerae* strains expressing differentially tagged HubP and MotW proteins were first grown in LB and whole cell lysates were subjected to SDS-PAGE and western blot analyses (see below). Multiple strains designed to express epitope-tagged MotV variants were also tested, however, no signal corresponding to a tagged MotV protein was detected.

The *V*. *cholerae* strain expressing HubP-3xFLAG and MotW-HA fusions from their native loci was grown in 2 L of LB at 37°C until OD_600 nm_ = 0.6. Cells were then centrifuged and resuspended in lysis buffer containing 20 mM HEPES pH 8.0, 100 mM NaCl, and cOmplete, EDTA-free protease inhibitors (Roche) and flash-frozen in liquid nitrogen. For membrane protein extraction, cells were thawed, lysed using an Emulsiflex C5 cell homogenizer (Avestin, Ottawa, Canada) and centrifuged at 12 000 x *g* for 15 min to remove cell debris. The supernatant was then subjected to ultracentrifugation using an SW28 Ti rotor at 26 500 rpm and 4°C for 1 h to pellet the membrane fraction. The latter was resuspended using a Potter-Elvehjem homogenizer in membrane resuspension buffer containing 20 mM HEPES pH 8.0, 120 mM NaCl, 10% glycerol, 5 mM MgCl_2_, 10 μM AppCp (Jena Bioscience, Jena, Germany), 2 μM c-di-GMP (MilliporeSigma, Burlington, MA) and 1 tablet per 50 mL cOmplete protease inhibitors. A mix of detergents was then added for membrane solubilization as follows: 0.4% w/v digitonin (MilliporeSigma), 0.4% w/v n-dodecyl-β-D-maltopyranoside (anagrade β-DDM, Anatrace, Maumee, OH), 0.4% w/v decyl maltose neopentyl glycol (DM-NPG, Anatrace), and 0.2% w/v lauryl maltose neopentyl glycol (LM-NPG, Anatrace). Following 1 h incubation at 18°C and under mild agitation, the solubilized membrane fraction was cleared by a second high-speed centrifugation as above. The supernatant was then incubated with anti-FLAG M2 affinity gel (100 μL resin/L of culture, MilliporeSigma), under mild agitation at 4°C for 1 h. After gravity elution of the non-bound fraction, the resin was washed extensively with affinity buffer containing all membrane resuspension buffer components and 0.008% w/v LM-NPG (>30 column bed volumes allowed to flow through the resin sequentially). The bound complexes were then eluted using four column bed volumes of elution buffer (affinity buffer supplemented with 3xFLAG peptide at 100 μg/mL), concentrated on a 100 kDa cut-off Amicon Ultra (MilliporeSigma) centrifugal filter and loaded on a 4–20% SDS-PAGE gradient gels for western blot analysis. Following gel electrophoresis, the migrated proteins were transferred onto 0.2 μm PVDF membranes (Amersham Hybond P, GE Healthcare, Chicago, IL) using a Trans-blot Turbo Transfer system (Bio-Rad, Hercules, CA). After a blocking step with 5% skim milk in TPBS (0.05% Tween-20 in 1× PBS), immunoblots were probed with mouse primary antibodies (anti-FLAG M2 antibody (dilution 1:1000, Sigma-Aldrich) or anti-HA antibody (dilution 1:1000, Thermo Fisher Scientific, Waltham, MA). Alexa-Fluor 680-conjugated donkey anti-mouse antibody (dilution 1:10,000, Abcam, Cambridge, United Kingdom) was used as secondary antibody. The signal was detected using a Li-Cor Odyssey Fc system (LI-COR Biosciences, Lincoln, NE) in the 700 nm channel.

## Supporting information

S1 Table Proteins showing enrichment in HubP+ minicells in iTRAQ experiments. (DOCX)

S2 Table Plasmids used in this study. (DOCX)

S3 Table Strains used in this study. (DOCX)

S4 Table Oligonucleotides used in this study. (DOCX)

S1 Data Numerical data for graphs. (XLSX)

S1 Fig Fluorescence-microscopy visualization of candidate polar proteins. Representative fluorescent microscopy images of C-terminal GFP fusions of candidate polar proteins in WT *V*. *cholerae*. _p_, plasmid-based expression. (A) Protein fusions showing diffuse distribution. (B) Protein fusions showing inclusion body formation. Inclusion bodies observed in the corresponding phase contrast images are indicated with arrowheads. Protein fusions exhibiting polar foci are shown in main Fig 1C. (C) Representative fluorescence microscopy images of GFP fusions of identified polar proteins (pseudocolored in green), coexpressed with HubP-PAmCherry (pseudocolored in red). Phase contrast images are also shown in blue. (D) VCA0220-GFP localization in *ΔparC V*. *cholerae* cells. Arrowheads indicate cells with diffuse fluorescence and without foci; arrows indicate slightly misplaced foci. Bars = 2 μm.
(TIF)

S2 Fig Characterization of mutants of newly identified polar proteins. (A) Growth rate measured by microplate reader. White bars in the left panel indicated transposon insertion mutants obtained from the defined *V*. *cholerae* transposon mutant library [31], derived from the C6706 strain. Grey bars in the right panel indicate clean in-frame deletion mutants obtained from the N16961 WT strain. Average and standard deviations of at least 3 independent experiments are shown. (B) Representative phase contrast images of indicated mutants. (C) Representative fluorescent microscopy images of HubP-GFP expressed from the plasmid in indicated mutants. (D) Representative negative-stain transmission electron microscopy images of indicated mutants. Numbers of flagella and bacteria detected in images were shown below. Bars, 2 μm (fluorescence microscopy); 1 μm (TEM).
(TIF)

S3 Fig Predicted topology and domain architectures of identified polar proteins. (A) Predicted protein folds and topologies. IM, inner membrane; PG, peptidoglycan. Protein fold prediction was carried out with Phyre2, Robetta, and AlphaFold2 and consensus protein folds were visualized with PyMOL (Schrödinger, LLC). Signal peptide detection was carried out with the SignalP-5.0 server. VC1210 and VC1380 do not feature detectable signal peptides for secretion or inner membrane targeting. (B) Predicted VCA0220/HlyB domain architecture. TM, transmembrane region; Tar, taxis towards aspartate; Tsr, taxis towards serine; LBD, ligand-binding domain; HAMP, histidine kinase-adenylate cyclase-methyl accepting protein-phosphatase domain; MA: methyl-accepting domain. Protein dimerization is based on multiple structures of conserved MCP proteins. (C) Predicted VC0632/DacB/PBP4 domain architecture. sp, signal peptide; D, domain. Protein dimerization is based on multiple structures of conserved DacB homologs. (D) Predicted VC1210 domain architecture. NTase-like, nucleotydiltransferase-like domain sharing predicted structural homology with the NTase domains of aminoglycoside adenyltransferases; DUF4111: α-helical domain of unknown function DUF4111/PF13427. (E) Representative growth curves of WT and *Δvc1210 V*. *cholerae* in the presence of the indicated concentrations of antibiotics. Km, kanamycin; Gm, gentamycin. OD_600_, optical density upon detection of transmitted light with 600 nm wavelength. Results are representative of three independent experiments. (F) VC1380 domain architecture. Primary sequence and fold prediction analyses do not detect conserved domains with significant confidence. (G) Predicted VC1909/MotV architecture. TPR, tetratricopeptide repeat; MIT, microtubule interacting and trafficking. (H) Predicted VC2232/MotW architecture. Ig, immunoglobulin; CC, coiled-coil; CT, C-terminus; K-rich, lysine-rich. Proposed protein dimerization is based on the presence of predicted coiled-coil regions in the protein, as well as on bacterial two-hybrid (BACTH) complementation assays (below). (I-K) Representative fluorescent microscopy images of plasmid-encoded, truncated VC1909/MotV (F) and VC2232/MotW (G and H) fused to GFP. The GFP moiety is expressed at the C-terminus of the indicated constructs. Bar = 2 μm.
(TIF)

S4 Fig Interactions among HubP, MotV and MotW. (A) Representative fluorescence microscopy images of plasmid-based MotV-GFP and MotW-GFP expression in Δ*motW* and Δ*motV* strain, respectively. Bar = 2 μm. (B) Chemotactic motility examined by swimming in soft agar plates. Average diameter relative to WT, along with standard deviations from three independent experiments are shown. (C) Western blot analyses of total cell lysates of *Vibrio cholerae* strains expressing differentially tagged HubP, MotW and MotV^$^ (^$^ undetected) proteins from the respective endogeneous chromosomal loci (_c_). Bio-Rad Precision Plus Protein molecular weight standards and bands specific to tagged HubP and MotW proteins are indicated. * denotes non-specifically reacted bands. (D) Anti-FLAG and anti-HA western blots of elution fractions from affinity pull-down experiments using anti-FLAG and anti-HA affinity resins and solubilized membrane extracts from *V*. *cholerae* cells co-expressing MotW-HA and HubP-FLAGx3 proteins. Theoretical molecular weights for the HubP and MotW proteins are also indicated. (E) BACTH analysis of the growth on an M9 maltose plate containing 5-Bromo-4-Chloro-3-Indolyl β-D-Galactopyranoside (X-gal). Representative results of 3 independent experiments were shown.
(TIF)

S5 Fig Motility defects examined by growth in soft agar. Complete graphs for motility defects examined by swimming in soft agar plates. Average diameter relative to WT, along with standard deviations from three or more experiments are shown. Δ*motV* and/or Δ*motW* strains are indicated in colored bars and *motV*^+^ *motW*^+^ strains are indicated in blank bars. * *motV*^*+*^ complemented in the suppressor strain.
(TIF)
