# Correction: Analysis of HubP-dependent cell pole protein targeting in *Vibrio cholerae* uncovers novel motility regulators

**DOI:** 10.1371/journal.pgen.1010094

**Published:** 2022-03-01

**Authors:** The PLOS Genetics Staff

The Editor’s affiliation is incorrect. The correct affiliation for editor Lotte Søgaard-Andersen is: Max Planck Institute for Terrestrial Microbiology, GERMANY. The publisher apologizes for the error.
