# Supplementary material for: Analysis of HubP-dependent cell pole protein targeting in Vibrio cholerae uncovers novel motility regulators
Source: PLoS Genet. 2022 Jan 12;18(1):e1009991. doi: 10.1371/journal.pgen.1009991 (PMC8789113; doi:10.1371/journal.pgen.1009991)
Supplement: S1 Table — (DOCX) [file pgen.1009991.s001.docx]

**S1 Table. Proteins showing enrichment in HubP^+^ minicells in iTRAQ experiments**

| **VC# ^a^** | **Description ^a,b^** | **Coverage (% protein)** | **Ratio ^c^** |
| --- | --- | --- | --- |
| VC1210 | hypothetical protein VC1210* | 6.8 | -1.66 |
| VC0998 | hypothetical protein VC0998^§^ | 1.2 | -1.64 |
| VCA1048 | Gfo/Idh/MocA family oxidoreductase* | 5.8 | -1.53 |
| VC1380 | hypothetical protein VC1380* | 9.5 | -1.49 |
| VC0928 | hypothetical protein VC0928* | 7.0 | -1.19 |
| VC2061 | ParA family protein^†^ | 27.1 | -1.06 |
| VCA0026 | hypothetical protein VCA0026* | 9.5 | -1.03 |
| VCA0773 | methyl-accepting chemotaxis protein^†^ | 14.6 | -0.99 |
| VC2059 | purine-binding chemotaxis protein CheW^†^ | 71.8 | -0.92 |
| VC1334 | 4-hydroxyphenylpyruvate dioxygenase* | 3.3 | -0.92 |
| VCA0663 | methyl-accepting chemotaxis protein^†^ | 16.7 | -0.91 |
| VCA0658 | methyl-accepting chemotaxis protein^†^ | 4.1 | -0.89 |
| VCA0906 | methyl-accepting chemotaxis protein^†^ | 26.1 | -0.88 |
| VC2232 | hypothetical protein VC2232* | 15.0 | -0.87 |
| VC1909 | hypothetical protein VC1909* | 27.5 | -0.83 |
| VC1406 | methyl-accepting chemotaxis protein^†^ | 4.6 | -0.83 |
| VCA0974 | methyl-accepting chemotaxis protein^†^ | 35.3 | -0.82 |
| VC2006 | chemotaxis protein CheV^†^ | 32.5 | -0.81 |
| VC1289 | methyl-accepting chemotaxis protein^†^ | 29.6 | -0.81 |
| VC0512 | methyl-accepting chemotaxis protein^†^ | 17.0 | -0.81 |
| VC2202 | chemotaxis protein CheV^†^ | 26.3 | -0.81 |
| VC1602 | chemotaxis protein CheV^†^ | 23.8 | -0.77 |
| VC0980 | epimerase* | 9.2 | -0.77 |
| VC2439 | methyl-accepting chemotaxis protein^†^ | 17.3 | -0.77 |
| VC1898 | methyl-accepting chemotaxis protein^†^ | 15.2 | -0.75 |
| VC1313 | methyl-accepting chemotaxis protein^†^ | 41.6 | -0.75 |
| VC2064 | chemotaxis protein CheZ^†^ | 35.2 | -0.75 |
| VC1362 | amino acid ABC transporter substrate-binding protein* | 12.6 | -0.75 |
| VC0737 | acetoin utilization protein AcuB* | 7.1 | -0.75 |
| VCA0954 | chemotaxis protein CheV^†^ | 37.3 | -0.74 |
| VC1349 | sensory box sensor histidine kinase/response regulator* | 0.5 | -0.73 |
| VC0678 | transcriptional activator HlyU* | 7.4 | -0.72 |
| VCA0068 | methyl-accepting chemotaxis protein^†^ | 11.9 | -0.71 |
| VC2060 | hypothetical protein VC2060^†^ | 8.0 | -0.70 |
| VCA0189 | response regulator* | 3.0 | -0.69 |
| VC0216 | methyl-accepting chemotaxis protein^†^ | 31.2 | -0.69 |
| VC2063 | chemotaxis protein CheA^†^ | 38.0 | -0.69 |
| VCA1034 | methyl-accepting chemotaxis protein^†^ | 16.7 | -0.65 |
| VCA1069 | methyl-accepting chemotaxis protein^†^ | 31.1 | -0.63 |
| VC2067 | MinD-like protein^†^ | 16.0 | -0.63 |
| VC1658 | serine transporter* | 4.8 | -0.62 |
| VC0632 | D-alanyl-D-alanine carboxypeptidase/endopeptidase* | 2.3 | -0.60 |
| VCA1033 | extracellular solute-binding protein* | 10.3 | -0.59 |
| VC2389 | carbamoyl phosphate synthase large subunit* | 3.5 | -0.57 |
| VCA0923 | methyl-accepting chemotaxis protein^†^ | 34.9 | -0.57 |
| VCA0220 | hemolysin secretion protein HylB^*^ | 25.9 | -0.56 |
| VCA0859 | aldo/keto reductase^*^ | 4.6 | -0.56 |
| VC1293 | aromatic amino acid aminotransferase* | 3.0 | -0.55 |
| VC0449 | methyl-accepting chemotaxis protein^†^ | 5.2 | -0.54 |
| VC0174 | hypothetical protein VC0174* | 21.6 | -0.52 |
| VCA0864 | methyl-accepting chemotaxis protein^†^ | 13.9 | -0.52 |

^a^ Based on the N16961 reference genome [1], NC_002505 and NC_002506

^b^ ^§^, HubP; ^†^, known chemotaxis-related proteins; *, proteins tested in this study

^c^ log2 of HubP^-^/HubP^+^ value from two samples

**Reference**

1. Heidelberg JF, Eisen JA, Nelson WC, Clayton RA, Gwinn ML, Dodson RJ, Haft DH, Hickey EK, Peterson JD, Umayam L, *et al* (2000) DNA sequence of both chromosomes of the cholera pathogen *Vibrio cholerae*. *Nature* 406: 477–83
