# Supplementary material for: Analysis of HubP-dependent cell pole protein targeting in Vibrio cholerae uncovers novel motility regulators
Source: PLoS Genet. 2022 Jan 12;18(1):e1009991. doi: 10.1371/journal.pgen.1009991 (PMC8789113; doi:10.1371/journal.pgen.1009991)
Supplement: S2 Table — (DOCX) [file pgen.1009991.s002.docx]

**S2 Table. Plasmids used in this study**

| **Plasmid** | **Description** |
| --- | --- |
| **Reference / construction*** | |
| ***I. Gene expression plasmids*** | |
| pBAD18kn | vector; pBRori, *P_ara_*, KmR |
| [1] | |
| pBAD33 | vector; P15Aori, *P_ara_*, CmR |
| [1] | |
| pEYY97 | cloning vector for C-terminal m-sf-GFP fusion |
| *m-sf-gfp* was amplified with oYo311 x oYo312 then cloned into SacI + XbaI sites of pBAD33 by ligation cloning. | |
| pEYY107 | pBAD33 *vc0998*(*hubP)-m-sf-gfp* |
| *hubP* fragment was obtained by NotI digestion of pYB470, and cloned into the NotI site of pEYY97 by ligation cloning. | |
| pEYY133 | cloning vector for C-terminal PAmCherry fusion |
| *PAmcherry* was amplified with oYo352 x oYo353 then cloned into SacI + XbaI sites of pBAD33 by ligation cloning. | |
| pEYY135 | pBAD33 *hubP-PAmcherry* |
| [2] | |
| pEYY146 | pBAD33 *vc0632(dacB)-m-sf-gfp* |
| *dacB(vc0632)* was amplified with oYo379 and oYo380 then cloned into pEYY97 digested by SacI and NotI. | |
| pEYY147 | pBAD33 *vc1909(motV)-m-sf-gfp* |
| *motV(vc1909)* was amplified with oYo395 and oYo396 then cloned into pEYY97 digested by SacI and NotI. | |
| pEYY148 | pBAD33 *vca0220(hlyB)-m-sf-gfp* |
| *hlyB(vca0220)* was amplified with oYo373 and oYo374 then cloned into pEYY97 digested by SacI and NotI. | |
| pEYY149 | pBAD33 *vc2232(motW)-m-sf-gfp* |
| *motW(vc2232)* was amplified with oYo397 and oYo398 then cloned into pEYY97 digested by SacI and NotI. | |
| pEYY152 | pBAD18kn *hubP-PAmCherry* |
| *hubP-PAmcherry* fragment was amplified with pBAD_F x pBAD_R from pEYY135 and cloned into the NheI + SphI sites of pBAD18kn. | |
| pEYY153 | pBAD33 *vc0678-m-sf-gfp* |
| *vc0678* was amplified with oYo406 and oYo407 then cloned into pEYY97 digested by SacI and NotI. | |
| pEYY185 | pBAD33 *motV* |
| *motV* was amplified with oYo395 and oYo465 then cloned into pBAD33 digested by SacI and XbaI. | |
| pEYY190 | pBAD33 *motW[∆cyto]-m-sf-gfp* |
| *motW*^1-396^ fragment was amplified with oYo397 and oYo466 then cloned into pEYY97 digested by SacI and NotI. | |
| pEYY199 | pBAD33 *motV[∆cyto]-m-sf-gfp* |
| *motV^1-51^* fragment was amplified with oYo395 and oYo527 then cloned into pEYY97 digested by SacI and NotI | |
| pEYY337 | pBAD33 *motV-cfp* |
| *motV* was amplified with oYo395 and oYo840 then cloned into pYB461 digested by SacI and NotI. | |
| pEYY338 | pBAD33 *motW-cfp* |
| *motW* was amplified with oYo397 and oYo841 then cloned into pYB461 digested by SacI and NotI. | |
| pEYY357 | pBAD33 *vc0737-m-sf-gfp* |
| *vc0737* was amplified with oYo381 and oYo385 then cloned into pEYY97 digested by SacI and NotI. | |
| pEYY359 | pBAD33 *vc0928-m-sf-gfp* |
| *vc0928* was amplified with oYo385 and oYo386 then cloned into pEYY97 digested by SacI and NotI | |
| pEYY361 | pBAD33 *vc1349-m-sf-gfp* |
| *vc1349* was amplified with oYo389 and oYo390 then cloned into pEYY97 digested by SacI and NotI. | |
| pEYY362 | pBAD33 *vc1362-m-sf-gfp* |
| *vc1362* was amplified with oYo391 and oYo392 then cloned into pEYY97 digested by SacI and NotI. | |
| pEYY363 | pBAD33 *vc1658-m-sf-gfp* |
| *vc1658* was amplified with oYo394 and oYo393 then cloned into pEYY97 digested by SacI and NotI | |
| pEYY365 | pBAD33 *vca0859-m-sf-gfp* |
| *vca0859* was amplified with oYo377 and oYo378 then cloned into pEYY97 digested by SacI and NotI. | |
| pEYY387 | pBAD33 *motW^1-374^-m-sf-gfp* |
| *motW^1-374^* fragment was amplified with oYo1056 and oYo1057 then cloned into pEYY97 digested by SacI and NotI. | |
| pEYY455 | pBAD33 *vc0174-m-sf-gfp* |
| *vc0174* was amplified with oY1381 and oYo1382 then cloned into pEYY97 digested by SacI and NotI. | |
| pEYY456 | pBAD33 *vc0980-m-sf-gfp* |
| *vc0980* was amplified with oY1369 and oYo1370 then cloned into pEYY97 digested by SacI and NotI. | |
| pEYY457 | pBAD33 *vc1210-m-sf-gfp* |
| *vc1210* was amplified with oY1369 and oYo1370 then cloned into pEYY97 digested by SacI and NotI. | |
| pEYY458 | pBAD33 *vc1293-m-sf-gfp* |
| *vc1293* was amplified with oY1379 and oYo1380 then cloned into pEYY97 digested by SacI and NotI. | |
| pEYY459 | pBAD33 *vc1334-m-sf-gfp* |
| *vc1334* was amplified with oY1373 and oYo1374 then cloned into pEYY97 digested by SacI and NotI. | |
| pEYY460 | pBAD33 *vc1380-m-sf-gfp* |
| *vc1380* was amplified with oY1371 and oYo1372 then cloned into pEYY97 digested by SacI and NotI. | |
| pEYY461 | pBAD33 *vc2389-m-sf-gfp* |
| *vc2389* was amplified with oY1377 and oYo1378 then cloned into pEYY97 digested by SacI and NotI. | |
| pEYY462 | pBAD33 *vca0026-m-sf-gfp* |
| *vca0026* was amplified with oY1385 and oYo1386 then cloned into pEYY97 digested by SacI and NotI. | |
| pEYY463 | pBAD33 *vca0189-m-sf-gfp* |
| *vca0189* was amplified with oY1387 and oYo1388 then cloned into pEYY97 digested by SacI and NotI. | |
| pEYY465 | pBAD33 *vca1048-m-sf-gfp* |
| *vca1048* was amplified with oY1383 and oYo1384 then cloned into pEYY97 digested by SacI and NotI. | |
| pYB461 | cloning vector for C-terminal CFP fusion |
| [3] | |
| pYB470 | pBAD33 *hubP-yfp* |
| [3] | |
| pYB484 | cloning vector for C-terminal mCherry fusion |
| [3] | |
| ***II. Allelic exchange plasmids*** | |
| pCVD442 | cloning vector; R6Kori, AmpR, sucB |
| [4] | |
| pCVD442∆parA1 | for *∆parA1* |
| [5] | |
| pEYY151 | for *∆dacB* |
| Upstream and downstream regions of *dacB* were amplified with oYo898 x oYo899 and oYo900 x oYo901, respectively. The two fragments were assembled into the SmaI site of pCVD442. | |
| pEYY155 | for *∆motV* |
| Upstream and downstream regions of *motV* were amplified with oYo909 x oYo910 and oYo911 x oYo912, respectively. Two fragments were assembled into the SmaI site of pCVD442. | |
| pEYY157 | for *∆motW* |
| Upstream and downstream regions of *motW* were amplified with oYo920 x oYo921 and oYo922 x oYo923, respectively. The two fragments were assembled into the SmaI site of pCVD442. | |
| pEYY158 | for *∆hlyB* |
| Upstream and downstream regions of *hlyB* were amplified with oYo931 x oYo932 and oYo933 x oYo934, respectively. The two fragments were assembled into the SmaI site of pCVD442. | |
| pEYY253 | for *motV^+^* |
| *motV* and its flanking regions were amplified with oYo909 and oYo910 then cloned into the SmaI site of pCVD442. | |
| pEYY274 | for *vieS*^H1002Y^ |
| DNA fragment containing H1002Y mutation of *vieS* was amplified with oYo613 x oYo618 then cloned into the SmaI site of pCVD442. | |
| pEYY273 | for *vieS*^T1030I^ |
| DNA fragment containing T1030I mutation of *vieS* was amplified with oYo613 x oYo618 then cloned into the SmaI site of pCVD442. | |
| pEYY275 | for *vieS*^A669V^ |
| DNA fragment containing A669V mutation of *vieS* was amplified with oYo613 x oYo618 then cloned into the SmaI site of pCVD442. | |
| pEYY281 | for *cheV4^1-161^* |
| DNA fragment containing *cheV4^1-161^* was amplified with oYo631 x oYo636 then cloned into the SmaI site of pCVD442. | |
| pEYY282 | for *cheV4*^E169K^ |
| DNA fragment containing E169K mutation of *cheV4* was amplified with oYo631 x oYo636 then cloned into the SmaI site of pCVD442. | |
| pEYY283 | for *cheV4*^T30A^ |
| DNA fragment containing T30A mutation of *cheV4* was amplified with oYo631 x oYo636 then cloned into the SmaI site of pCVD442. | |
| pEYY286 | for *∆fliG* |
| Upstream and downstream regions of *fliG* were amplified with oYo619 x oYo620 and oYo623 x oYo624, respectively. The two fragments were assembled into the SmaI site of pCVD442. | |
| pEYY287 | for *∆fliM* |
| Upstream and downstream regions of *fliM* were amplified with oYo625 x oYo655 and oYo656 x oYo630, respectively. The two fragments were assembled into the SmaI site of pCVD442. | |
| pEYY288 | for *∆cheV4* |
| Upstream and downstream regions of *cheV4* were amplified with oYo631 x oYo701 and oYo636 x oYo702, respectively. The two fragments were assembled into the SmaI site of pCVD442. | |
| pEYY289 | for *∆vc1653(vieS)* |
| Upstream and downstream regions of *vieS* were amplified with oYo613 x oYo699 and oYo618 x oYo700, respectively. The two fragments were assembled into the SmaI site of pCVD442. | |
| pEYY293 | for *hubP-FLAGx3* |
| DNA fragments containing 3’-end of *hubP* and the downstream region of *hubP* were amplified with oYo721 x oYo728, and oYo729 x oYo573, respectively. The two fragments were assembled into the SmaI site of pCVD442. | |
| pEYY301 | for *motW-FLAGx3* |
| DNA fragments containing 3’-end of *motW* and the downstream region of *motW* were amplified with oYo928 x oYo768, and oYo920 x oYo769, respectively. The two fragments were assembled into the SmaI site of pCVD442. | |
| pEYY302 | for *HA-motV* |
| DNA fragments containing 5' of *motV* and the upstream region of *motV* were amplified with oYo832 x oYo833, and oYo834 x oYo912, respectively. The two fragments were assembled into the SmaI site of pCVD442. | |
| pEYY306 | for *fliM*^D180N^ |
| DNA fragment containing D180N mutation of *fliM* was amplified with oYo625 x oYo630 then cloned into the SmaI site of pCVD442. | |
| pEYY307 | for *fliM*^G111E^ |
| DNA fragment containing G111E mutation of *fliM* was amplified with oYo625 x oYo630 then cloned into the SmaI site of pCVD442. | |
| pEYY308 | for *fliM*^R107C^ |
| DNA fragment containing R107C mutation of *fliM* was amplified with oYo625 x oYo630 then cloned into the SmaI site of pCVD442. | |
| pEYY316 | for *fliG*^A59S^ |
| DNA fragment containing A59S mutation of *fliG* was amplified with oYo619 x oYo624 then cloned into the SmaI site of pCVD442. | |
| pEYY317 | for *fliG*^K281E^ |
| DNA fragment containing K281E mutation of *fliG* was amplified with oYo619 x oYo624 then cloned into the SmaI site of pCVD442. | |
| pEYY318 | for *fliG*^F199L^ |
| DNA fragment containing F199L mutation of *fliG* was amplified with oYo619 x oYo624 then cloned into the SmaI site of pCVD442. | |
| pEYY320 | for *fliG*^G119D^ |
| DNA fragment containing G119D mutation of *fliG* was amplified with oYo619 x oYo624 then cloned into the SmaI site of pCVD442. | |
| pEYY321 | for *motV-eCFP* |
| DNA fragment containing *cfp* was amplified with oYo785 x oYo811 then assembled in pCVD442 containing *motV-GFP* amplified by oYo469 x oYo786. | |
| pEYY322 | for *motW-eCFP* |
| DNA fragment containing *cfp* was amplified with oYo814 x oYo815 then assembled in pCVD442 containing *motW* amplified by oYo812 x oYo813. | |
| pEYY333 | for *motW-HA* |
| DNA fragments containing 3’-end of *motW* and the downstream region of *motW* were amplified with oYo928 x oYo830, and oYo920 x oYo831, respectively. The two fragments were assembled into the SmaI site of pCVD442. | |
| pEYY334 | for *hubP-HA* |
| DNA fragments containing 3’-end of *hubP* and the downstream region of *hubP* were amplified with oYo721 x oYo828, and oYo573 x oYo829, respectively. The two fragments were assembled into the SmaI site of pCVD442. | |
| pEYY335 | for *FLAGx3-motV* |
| DNA fragments containing 5' of *motV* and the upstream region of *motV* were amplified with oYo832 x oYo835, and oYo836 x oYo912, respectively. The two fragments were assembled into the SmaI site of pCVD442. | |
| pEYY383 | for *cheV4-gfp* |
| DNA fragments containing *cheV4-gfp* and the downstream region of *cheV4* were amplified with oYo1052 x oYo1053, and oYo636 x oYo1054, respectively. The two fragments were assembled into the SmaI site of pCVD442. | |
| pEYY389 | for *cheV4^1-161^-gfp* |
| DNA fragments containing *cheV4^1-161^-gfp* and the downstream region of *cheV4* were amplified with oYo1053 x oYo1060, and oYo636 x oYo1054, respectively. The two fragments were assembled into the SmaI site of pCVD442. | |
| pEYY466 | for *∆vc1210* |
| Upstream and downstream regions of *vc1210* were amplified with oYo1393 x oYo1394 and oYo1395 x oYo1396, respectively. The two fragments were assembled into the SmaI site of pCVD442. | |
| pEYY467 | for *∆1380* |
| Upstream and downstream regions of *vc1380* were amplified with oYo1397 x oYo1398 and oYo1399 x oYo1400, respectively. The two fragments were assembled into the SmaI site of pCVD442. | |
| pRB004 | for ∆*hubP* |
| [3] | |
| pYB213 | for *∆vc1960 (minD)* |
| Upstream and downstream regions of *minD* were amplified with YPR347 x YPR348 and YPR351 x YPR352, respectively. The two fragments were ligated at NheI site and cloned into the XbaI site of pCVD442. | |
| pYB517 | for *hubP::hubP-yfp* |
| [3] | |
| ***III. Bacterial Two Hybrid (BACTH) plasmids*** | |
| pKT25 | BACTH cloning vector; p15A ori, Km^R^ |
| Euromedex | |
| pK T25-hubP^peri^ | pKT25 *T25-hubP^46-284^* |
| DNA fragment encoding periplasmic region of HubP^46-284^ was amplified with oYo1308 x oYo1309 then cloned into XbaI-EcoRI sites of pKT25 by ligation cloning. | |
| pK T25-motW^peri^ | pKT25 *T25-motW^20-374^* |
| DNA fragment encoding periplasmic region of motW^20-374^ was amplified with oYo1326 x oYo1327 then cloned into KpnI-BamHI sites of pKT25 by ligation cloning. | |
| pKNT25-YY | BACTH cloning vector; p15A ori, Km^R^ |
| [3] | |
| pK hubP^peri^-T25 | pKNT25 *hubP^46-284^-T25* |
| DNA fragment encoding periplasmic region of HubP^46-284^ was amplified with oYo1306 x oYo1307 then cloned into NotI site of pKNT25-YY by ligation cloning. | |
| pUT18C | BACTH cloning vector; pUC ori, Amp^R^ |
| Euromedex | |
| pU T18-hubP^peri^ | pUT18C *T18-hubP^46-284^* |
| DNA fragment encoding periplasmic region of HubP^46-284^ was amplified with oYo1308 x oYo1309 then cloned into XbaI-EcoRI sites of pUT18C by ligation cloning. | |
| pU T18-motW^peri^ | pUT18C *T18- motW^20-374^* |
| DNA fragment encoding periplasmic region of motW^20-374^ was amplified with oYo1326 x oYo1327 then cloned into KpnI-BamHI sites of pUT18C by ligation cloning. | |
| pUT18-YY | BACTH cloning vector; pUC ori, Amp^R^ |
| [3] | |
| pU hubP^peri^-T18 | pUT18 *hubP^46-284^-T18* |
| DNA fragment encoding periplasmic region of HubP^46-284^ was amplified with oYo1306 x oYo1307 then cloned into NotI site of pUT18-YY by ligation cloning. | |

* Unless otherwise noted, Gibson assembly was used.

**References**

1. Guzman LM, Belin D, Carson MJ & Beckwith J (1995) Tight regulation, modulation, and high-level expression by vectors containing the arabinose PBAD promoter. *J Bacteriol* 177: 4121–30

2. Altinoglu I, Merrifield CJ & Yamaichi Y (2019) Single molecule super-resolution imaging of bacterial cell pole proteins with high-throughput quantitative analysis pipeline. *Sci Rep* 9: 6680

3. Yamaichi Y, Bruckner R, Ringgaard S, Möll A, Cameron DE, Briegel A, Jensen GJ, Davis BM & Waldor MK (2012) A multidomain hub anchors the chromosome segregation and chemotactic machinery to the bacterial pole. *Genes Dev* 26: 2348–60

4. Donnenberg MS & Kaper JB (1991) Construction of an *eae* deletion mutant of enteropathogenic *Escherichia coli* by using a positive-selection suicide vector. *Infect Immun* 59: 4310–7

5. Fogel MA & Waldor MK (2006) A dynamic, mitotic-like mechanism for bacterial chromosome segregation. *Genes Dev* 20: 3269–82
