# Supplementary material for: Analysis of HubP-dependent cell pole protein targeting in Vibrio cholerae uncovers novel motility regulators
Source: PLoS Genet. 2022 Jan 12;18(1):e1009991. doi: 10.1371/journal.pgen.1009991 (PMC8789113; doi:10.1371/journal.pgen.1009991)
Supplement: S3 Table — (DOCX) [file pgen.1009991.s003.docx]

**S3 Table. Strains used in this study**

| **Strain** | **Description** | **Reference** |
| --- | --- | --- |
| ***Escherichia coli*** | | |
| DH5α | host strain of general cloning | laboratory stock |
| DH5αλ*pir* | host strain for the cloning of R6K*ori* plasmid | laboratory stock |
| SM10 λ*pir* | host strain for conjugation | laboratory stock |
| BTH101 | Host strain for bacterial two hybrid | laboratory stock |
| JW3778 | *∆cyaA::kan* | [1] |
| bEYY2122 | BTH101 *∆cyaA* | This study |
| ***Vibrio cholerae*** | | |
| C6706 | *V. cholerae* O1 El Tor, *hapR^+^,* SmR | [2] |
| 5-G5 | C6706 *dacB::Tn* | [2] |
| 3-D7 | C6706 *vc1210::Tn* | [2] |
| 8-G1 | C6706 *vc1380::Tn* | [2] |
| 4-H10 | C6706 *motV::Tn* | [2] |
| 9-B3 | C6706 *motW::Tn* | [2] |
| 2-H9 | C6706 *hlyB::Tn* | [2] |
| N16961 | *V. cholerae* O1 El Tor, SmR | [3] |
| N16961 ∆*parC* | N16961 *∆parC* | S. Ringgaard |
| MKW1383 | N16961 *∆ctxAB::kan* | [4] |
| bEYY1014 | N16961 *∆ctxAB::kan ∆hubP* | This study |
| bEYY1319 | N16961 *∆minD ∆parA1 ∆hubP* | This study |
| bEYY1402 | N16961 *∆ctxAB::kan ∆dacB* | This study |
| bEYY1415 | N16961 *∆ctxAB::kan ∆motV* | This study |
| bEYY1422 | N16961 *∆ctxAB::kan ∆motW* | This study |
| bEYY1427 | N16961 *∆ctxAB::kan ∆hlyB* | This study |
| bEYY1436 | N16961 *∆ctxAB::kan ∆motW* / pEYY147 | This study |
| bEYY1439 | N16961 *∆ctxAB::kan ∆motV* / pEYY149 | This study |
| bEYY1467 | N16961 *∆motV ∆motW* | This study |
| bEYY1614 | N16961 *∆ctxAB::kan* / pEYY199 | This study |
| bEYY1802 | N16961 *cheV*^1-161^ | This study |
| bEYY1803 | N16961 *cheV*^E169K^ | This study |
| bEYY1804 | N16961 *cheV*^T30A^ | This study |
| bEYY1805 | N16961 *vieS*^T1030I^ | This study |
| bEYY1806 | N16961 *vieS*^H1002Y^ | This study |
| bEYY1807 | N16961 *vieS*^A669V^ | This study |
| bEYY1816 | N16961 *∆fliM* | This study |
| bEYY1817 | N16961 *∆cheV* | This study |
| bEYY1818 | N16961 *∆vieS* | This study |
| bEYY1820 | N16961 ∆*motV* | This study |
| bEYY1821 | N16961 ∆*motW* | This study |
| bEYY1851 | N16961 *vieS*^T1030I^ *∆motW* | This study |
| bEYY1848 | N16961 *cheV*^1-161^ ∆*motV* | This study |
| bEYY1849 | N16961 *cheV*^E169K^ *∆motV* | This study |
| bEYY1850 | N16961 *cheV*^T30A^ *∆motV* | This study |
| bEYY1852 | N16961 *vieS*^H1002Y^ *∆motW* | This study |
| bEYY1853 | N16961 *vieS*^A669V^ *∆motW* | This study |
| bEYY1867 | C6706 *hubP-FLAGx3* | This study |
| bEYY1895 | N16961 *∆fliG* | This study |
| bEYY1927 | N16961 *fliG*^A59S^ | This study |
| bEYY1928 | N16961 *fliG*^K281E^ | This study |
| bEYY1929 | N16961 *fliG*^F199L^ | This study |
| bEYY1931 | N16961 *fliG*^G119D^ | This study |
| bEYY1932 | N16961 *fliG*^A59S^ *∆motV* | This study |
| bEYY1933 | N16961 *fliG*^K281E^ *∆motV* | This study |
| bEYY1934 | N16961 *fliG*^F199L^ ∆*motV* | This study |
| bEYY1935 | N16961 *fliG*^G119D^ ∆*motV* | This study |
| bEYY1943 | N16961 *hubP-yfp motV-cfp* | This study |
| bEYY1944 | N16961 *hubP-yfp motW-cfp* | This study |
| bEYY1945 | N16961 *fliM*^D180N^ | This study |
| bEYY1946 | N16961 *fliM*^G111E^ | This study |
| bEYY1947 | N16961 *fliM*^R107C^ | This study |
| bEYY1948 | N16961 *fliM*^D180N^ ∆*motV* | This study |
| bEYY1949 | N16961 *fliM*^G111E^ *∆motV* | This study |
| bEYY1950 | N16961 *fliM*^R107C^ *∆motV* | This study |
| bEYY1966 | C6706 *hubP-FLAGx3* HA-*motV* | This study |
| bEYY1967 | C6706 *hubP-FLAGx3* *motW-HA* | This study |
| bEYY1971 | C6706 *motW-HA* | This study |
| bEYY1974 | C6706 *hubP-HA FLAGx3-motV* | This study |
| bEYY1975 | C6706 *hubP-HA motW-FLAGx3* | This study |
| bEYY1995 | N16961 *hubP-yfp* / pEYY337 | This study |
| bEYY1996 | N16961 *hubP-yfp* / pEYY338 | This study |
| bEYY2055 | N16961 *fliM*^D180N^ *fliG*^G119D^ ∆*motV* | This study |
| bEYY2069 | N16961 *fliM*^R107C^ *fliG*^A59S^ *∆motV* | This study |
| bEYY2109 | N16961 ∆*motV* / pEYY149 | This study |
| bEYY2113 | N16961 *∆motW* / pEYY190 | This study |
| bEYY2145 | N16961 *∆motW* / pEYY387 | This study |
| bEYY2148 | N16961 *cheV4-gfp* | This study |
| bEYY2173 | N16961 ∆*hubP* *cheV4-gfp* | This study |
| bEYY2174 | N16961 *cheV4^1-161^-gfp* | This study |
| bEYY2323 | N16961 *fliM*^D180N^ *fliG*^G119D^ | This study |
| bEYY2347 | N16961 ∆*hubP* *cheV4^1-161^-gfp* | This study |
| bEYY2350 | N16961 ∆*parC* *cheV4-gfp* | This study |
| bEYY2351 | N16961 ∆*parC cheV4^1-161^-gfp* | This study |
| bEYY2455 | N16961 *∆motV ∆motW fliM^D180N^ fliG^G119D^* | This study |
| bEYY2456 | N16961 *∆hubP ∆motV fliM^D180N^ fliG^G119D^* | This study |
| bEYY2465 | N16961 *∆hubP fliM^D180N^ fliG^G119D^* | This study |
| bEYY2490 | N16961 *∆motW* / pEYY185 | This study |
| bEYY2524 | N16961 *∆vc1210* | This study |
| bEYY2525 | N16961 *∆vc1380* | This study |
| YBB438 | N16961 *∆minD ∆parA1* | This study |
| YBB2011 | N16961 *∆hubP* | [5] |
| YBB2413 | N16961 *∆minD ∆parA1 hubP-yfp* | This study |
| A1 | N16961 *∆ctxAB::kan ∆motV suppressor mutations* (see table 1) | This study |
| A1 *motV+* | A1 but *motV+* | This study |
| A2 | N16961 *∆ctxAB::kan ∆motV suppressor mutations (*see table 1) | This study |
| A2 *motV+* | A1 but *motV+* | This study |
| A3 | N16961 *∆ctxAB::kan ∆motV suppressor mutations* (see table 1) | This study |
| A3 *motV+* | A3 but *motV+* | This study |
| A4 | N16961 *∆ctxAB::kan ∆motV suppressor mutations (*see table 1) | This study |
| A4 *motV+* | A4 but *motV*+ | This study |
| A6 | N16961 *∆ctxAB::kan ∆motV suppressor mutations* (see table 1) | This study |
| A6 *motV+* | A6 but *motV+* | This study |
| A8 | N16961 *∆ctxAB::kan ∆motV suppressor mutations* (see table 1) | This study |
| A8 *motV+* | A8 but *motV+* | This study |
| B1 | N16961 *∆ctxAB::kan ∆motW suppressor mutations* (see table 1) | This study |
| B1 *motW+* | B1 but *motW*+ | This study |
| B2 | N16961 *∆ctxAB::kan ∆motW suppressor mutations* (see table 1) | This study |
| B2 *motW+* | B2 but *motW*+ | This study |
| B4 | N16961 *∆ctxAB::kan ∆motW suppressor mutations* (see table 1) | This study |
| B4 *motW+* | B4 but *motW*+ | This study |
| B6 | N16961 *∆ctxAB::kan ∆motW suppressor mutations* (see table 1) | This study |
| B6 *motW+* | B6 but *motW*+ | This study |
| B7 | N16961 *∆ctxAB::kan ∆motW suppressor mutations* (see table 1) | This study |
| B7 *motW+* | B7 but *motW*+ | This study |
| B8 | N16961 *∆ctxAB::kan ∆motW suppressor mutations* (see table 1) | This study |
| B8 *motW*+ | B8 but *motW+* | This study |

**References**

1. Baba T, Ara T, Hasegawa M, Takai Y, Okumura Y, Baba M, Datsenko KA, Tomita M, Wanner BL & Mori H (2006) Construction of *Escherichia coli* K-12 in-frame, single-gene knockout mutants: the Keio collection. *Mol Syst Biol* 2: 2006.0008

2. Cameron DE, Urbach JM & Mekalanos JJ (2008) A defined transposon mutant library and its use in identifying motility genes in *Vibrio cholerae*. *Proc Natl Acad Sci U S A* 105: 8736–41

3. Heidelberg JF, Eisen JA, Nelson WC, Clayton RA, Gwinn ML, Dodson RJ, Haft DH, Hickey EK, Peterson JD, Umayam L, *et al* (2000) DNA sequence of both chromosomes of the cholera pathogen *Vibrio cholerae*. *Nature* 406: 477–83

4. Altinoglu I, Merrifield CJ & Yamaichi Y (2019) Single molecule super-resolution imaging of bacterial cell pole proteins with high-throughput quantitative analysis pipeline. *Sci Rep* 9: 6680

5. Yamaichi Y, Bruckner R, Ringgaard S, Möll A, Cameron DE, Briegel A, Jensen GJ, Davis BM & Waldor MK (2012) A multidomain hub anchors the chromosome segregation and chemotactic machinery to the bacterial pole. *Genes Dev* 26: 2348–60
