# Supplementary material for: Analysis of HubP-dependent cell pole protein targeting in Vibrio cholerae uncovers novel motility regulators
Source: PLoS Genet. 2022 Jan 12;18(1):e1009991. doi: 10.1371/journal.pgen.1009991 (PMC8789113; doi:10.1371/journal.pgen.1009991)
Supplement: S4 Table — (DOCX) [file pgen.1009991.s004.docx]

**S4 Table. Oligonucleotides used in this study**

| **Name** | **Sequence (5'-3')** |
| --- | --- |
| oYo78 | GCTGAATGGGCGAGTGGGTGGTGCGGCCGCCGGTGGCAGTAAAGGTGAAGAAC |
| oYo79 | GTCGAAAGAATCAGAGGGAGGCTAGCTTATTTGTAGAGTTCATCCATG |
| oYo93 | GCTGAATGGGCGAGTGGGTGGTGCGGCCGCCGGTGGCAGTGTGATTAAACCAGACATG |
| oYo94 | GTCGAAAGAATCAGAGGGAGGCTAGCTTACTTGGCCTGCCTCGGCAG |
| oYo311 | AGCTGAGCTCAAGGAGGAGCGGCCGCCGTGAGTAAAGGTGAAGAACTGTTC |
| oYo312 | GCTCTAGATTATTTGTAGAGTTCATCCATGC |
| oYo352 | AGCTGAGCTCAAGGAGGAGCGGCCGCCGTGAGCAAGGGCGAGGAG |
| oYo353 | GCTCTAGATTACTTGTACAGCTCGTCCATGC |
| oYo373 | TGGGCTAGCGAATTCGAGCTATGATCATCAATAAATTTTCCCTTAAATGG |
| oYo374 | CACCTTTACTCACGGCGGCCGCAAGTTCAAAACTGCCCACC |
| oYo377 | TGGGCTAGCGAATTCGAGCTATGCAACAACAAGCAACCGTAC |
| oYo378 | CACCTTTACTCACGGCGGCCGCTGGCACGCCATGACCTTTG |
| oYo379 | TGGGCTAGCGAATTCGAGCTTTGGTGGATGTTATTGCTATGC |
| oYo380 | CACCTTTACTCACGGCGGCCGCGTTTGGCTTAGATGCCAAACGA |
| oYo381 | TGGGCTAGCGAATTCGAGCTATGGGAAAAGGGGTTAGAGAC |
| oYo385 | TGGGCTAGCGAATTCGAGCTTTGTCTAACTTTAAAGGATCTATC |
| oYo386 | CACCTTTACTCACGGCGGCCGCTTTTTTTACCACTGTCATTGACTG |
| oYo389 | TGGGCTAGCGAATTCGAGCTATGCGCTCGATAAAAGCG |
| oYo390 | CACCTTTACTCACGGCGGCCGCCTGGTTTAAACGCTCACCG |
| oYo391 | TGGGCTAGCGAATTCGAGCTATGGCGAATAAACTCACTGTTCTTG |
| oYo392 | CACCTTTACTCACGGCGGCCGCACGGATAGGTGGTGCGTAC |
| oYo393 | TGGGCTAGCGAATTCGAGCTGTGAAAGACTCTCAGGAAGTG |
| oYo394 | CACCTTTACTCACGGCGGCCGCGCGAAGTAAACTGTAGAGCAG |
| oYo395 | TGGGCTAGCGAATTCGAGCTTTGAAAACTGAAGATACGGATTTTATG |
| oYo396 | CACCTTTACTCACGGCGGCCGCCCACTTTTTCTTCTCGCCAAAG |
| oYo397 | TGGGCTAGCGAATTCGAGCTATGTTGAGGATTCTCGCGG |
| oYo398 | CACCTTTACTCACGGCGGCCGCCTTCCCCTTCTTAGGCGG |
| oYo406 | CACCTTTACTCACGGCGGCCGCCTGATTCGCCTGACAATAAAGAC |
| oYo407 | TGGGCTAGCGAATTCGAGCTATGCCGTATTTAAAGGGGGC |
| oYo465 | GCCTGCAGGTCGACTCTAGATTACCACTTTTTCTTCTCGCC |
| oYo469 | CCTTGCTCACCATGGCGGCCGCCCACTTTTTCTTCTCGCCAAAG |
| oYo527 | CACCTTTACTCACGGCGGCCGCGGCCACATAGCGCGAGGATTC |
| oYo573 | CGGATAACAATTTGTGGAATTCCCACGATAACGGCGTGCAGTGGC |
| oYo613 | ATGCGATATCGAGCTCTCCCTTAACACCTTAATCCGATGAAAACTGTCCTGA |
| oYo618 | TAACAATTTGTGGAATTCCCCGTACTAGCGCTTCAACGCC |
| oYo619 | ATGCGATATCGAGCTCTCCCCAAGAGTCGTTCCAGCGTCA |
| oYo620 | TCACCAGGCCTTAGCCATTTTGCATC |
| oYo623 | GTTCTTCTGATCAAGTAGGATCGGTGT |
| oYo624 | TAACAATTTGTGGAATTCCCGCTTTTCGACCTGAGCATTAAGCAG |
| oYo625 | ATGCGATATCGAGCTCTCCCAATGTGGCAACAAAGCGTGATG |
| oYo630 | TAACAATTTGTGGAATTCCCCAAAAGCGACCCCAAGGTGG |
| oYo631 | ATGCGATATCGAGCTCTCCCCACATTTGCGGTCAACACCACT |
| oYo636 | TAACAATTTGTGGAATTCCCAATGAGCTGCCTGAGCTAAATACT |
| oYo655 | TTTTCCTTTTGCGGCCGCATTTCACCTATTGCATTACAAAGTCG |
| oYo656 | GCGGCCGCAAAAGGAAAATCCGTTTTACGACGTCGG |
| oYo699 | TTTCATAACGAAATAATGCAAAAACACTTAACAGTAATGTAAAGAAATATCG |
| oYo700 | TGCATTATTTCGTTATGAAAATAATGATAGTAGAAGATGATCGAATTCAGG |
| oYo701 | ACTTAACCGAAATATTTTCTTTAACAGTTCGGACTCTTACTTATACTAAACG |
| oYo702 | AGAAAATATTTCGGTTAAGTATTTAACTCAAAGCCAATCGC |
| oYo721 | CCGCATGCGATATCGAGCTCTCCCAGATCAGCAAGCGCAGTTG |
| oYo728 | ATGTCATGGTCCTTATAGTCTCCGTCGTGGTCTTTGTAGTCTCCCGAAGATGCACTACCCACTCGCCCATTCAGCACATC |
| oYo729 | AAGACCACGACGGAGACTATAAGGACCATGACATCGATTACAAAGACGATGATGATAAGTAGCTCCCTCTGATTCTTTCG |
| oYo768 | ATGTCATGGTCCTTATAGTCTCCGTCGTGGTCTTTGTAGTCTCCCGAAGATGCACTACCCTTCCCCTTCTTAGGCGGCAT |
| oYo769 | AAGACCACGACGGAGACTATAAGGACCATGACATCGATTACAAAGACGATGATGATAAGTAGAGGCGTTGAATTTGCCTT |
| oYo785 | CAAGTCATTATCGTGCGGTATCTTACTTGTACAGCTCGTCCATG |
| oYo786 | CATGGACGAGCTGTACAAGTAAGATACCGCACGATAATGACTTG |
| oYo811 | CGAGAAGAAAAAGTGGGCGGCCGCCATGGTGAGCAAGGGCGAG |
| oYo812 | CATGGACGAGCTGTACAAGTAAAGGCGTTGAATTTGCCTTTGACC |
| oYo813 | CTCGCCCTTGCTCACCATGGCGGCCGCCTTCCCCTTC |
| oYo814 | GAAGGGGAAGGCGGCCGCCATGGTGAGCAAGGGCGAG |
| oYo815 | GGTCAAAGGCAAATTCAACGCCTTTACTTGTACAGCTCGTCCATG |
| oYo828 | TCATACGGATAACCGCTGCTTGCGCTACCCACTCGCCCATTCAGCACATC |
| oYo829 | GTTATCCGTATGATGTTCCGGATTATGCATAGCTCCCTCTGATTCTTTCG |
| oYo830 | GTTATCCGTATGATGTTCCGGATTATGCATAGAGGCGTTGAATTTGCCTT |
| oYo831 | TCATACGGATAACCGCTGCTTGCGCTACCCTTCCCCTTCTTAGGCGGCAT |
| oYo832 | CCGCATGCGATATCGAGCTCTCCCCGAATCTGAATGGTCTCTAAGC |
| oYo833 | GTTCCGGATTATGCAGGTAGCGCAAGCAGCGGTAAAACTGAAGATACGGATTTTATGAAT |
| oYo834 | TTGCGCTACCTGCATAATCCGGAACATCATACGGATACAATGGCTAACGATTAAGGATAC |
| oYo835 | ATAAGGACCATGACATCGATTACAAAGACGATGATGATAAGGGTAGTGCATCTTCGGGAAAAACTGAAGATACGGATTTTATGAAT |
| oYo836 | TCATCGTCTTTGTAATCGATGTCATGGTCCTTATAGTCTCCGTCGTGGTCTTTGTAGTCCAATGGCTAACGATTAAGGATAC |
| oYo840 | TTGCTCACCATGGCGGCCGCCCACTTTTTCTTCTCGCCAAAG |
| oYo841 | TTGCTCACCATGGCGGCCGCCTTCCCCTTCTTAGGCGG |
| oYo898 | CCGCATGCGATATCGAGCTCTCCCTGATGTGGAGCTACTACGAGC |
| oYo899 | CTTGGTGGATGTTATTGCTATGCCTGAAAAATAAAAAGCGCCG |
| oYo900 | CGGCGCTTTTTATTTTTCAGGCATAGCAATAACATCCACCAAG |
| oYo901 | CGGATAACAATTTGTGGAATTCCCTGTCTGCTATCTACACTTTCG |
| oYo909 | CCGCATGCGATATCGAGCTCTCCCCGGCTAGCCGGAATTG |
| oYo910 | CATTGAAAACTGAAGATACGGATTTTATGAATATAGCCGCACGATAATGACTTGATTAAC |
| oYo911 | GTTAATCAAGTCATTATCGTGCGGCTATATTCATAAAATCCGTATCTTCAGTTTTCAATG |
| oYo912 | CGGATAACAATTTGTGGAATTCCcGCAACCGCCTTGATGC |
| oYo920 | CCGCATGCGATATCGAGCTCTCCCCGCGCAGATCTTGCAAAC |
| oYo921 | GTTATGTTGAGGATTCTCGCGGGCGTTGAATTTGCCTTTGACC |
| oYo922 | GGTCAAAGGCAAATTCAACGCCCGCGAGAATCCTCAACATAAC |
| oYo923 | CGGATAACAATTTGTGGAATTCCCCCCGAGTACAAAGATGTCCATG |
| oYo928 | CGGATAACAATTTGTGGAATTCCCGGAGAGCAGGGGATGATTG |
| oYo931 | CCGCATGCGATATCGAGCTCTCCCGGTCAGTGATTGTGGAATGCC |
| oYo932 | GACATGATCATCAATAAATTTTCCCTTAAATGGCAATGACTACGCTAAGCAAGCATTC |
| oYo933 | GAATGCTTGCTTAGCGTAGTCATTGCCATTTAAGGGAAAATTTATTGATGATCATGTC |
| oYo934 | CGGATAACAATTTGTGGAATTCCCGGTGATTTCCAATATGGCGGTC |
| oYo1056 | CACCTTTACTCACGGCGGCCGCGTTGCGGCTACGCTGCTCTT |
| oYo1057 | TTGGGCTAGCGAATTCGAGCTCATGTTGAGGATTCTCGCGGC |
| oYo1306 | AATTGCGGCCGCGCAGAAAGCATTCGTCTTG |
| oYo1307 | AATTGCGGCCGCATCTAAAGCTGATGGCGC |
| oYo1308 | TTAATCTAGAGGCAGAAAGCATTCGTCTTG |
| oYo1309 | AATTGAATTCTAATCTAAAGCTGATGGCGC |
| oYo1326 | TTAAGGATCCGGCACAACAATCTAGCA |
| oYo1327 | TTAAGGTACCTTAGTTGCGGCTACGCTGC |
| oYo1369 | TGGGCTAGCGAATTCGAGCTGTGCAAATTAAACGAGTATGG |
| oYo1370 | CACCTTTACTCACGGCGGCCGCACCAATTCGAAACTCGGTT |
| oYo1371 | TGGGCTAGCGAATTCGAGCTGTGAAAGCCTCTGAACTG |
| oYo1372 | CACCTTTACTCACGGCGGCCGCCTCTTCAAGCATAGTACCTAAAAT |
| oYo1373 | TGGGCTAGCGAATTCGAGCTATGCTGAAGTTTTTTAAACCCTC |
| oYo1374 | CACCTTTACTCACGGCGGCCGCCTTCAAGAAGCCGAGTTC |
| oYo1375 | TGGGCTAGCGAATTCGAGCTATGAGAATTTTAATTACTGGAGG |
| oYo1376 | CACCTTTACTCACGGCGGCCGCTTGTGATGCCGTAAGGAG |
| oYo1377 | TGGGCTAGCGAATTCGAGCTATGCCAAAACGTACTGAC |
| oYo1378 | CACCTTTACTCACGGCGGCCGCAGCTTGGTTCGCTTTTAC |
| oYo1379 | TGGGCTAGCGAATTCGAGCTTTGATGAGTTCAATCATCCCTTC |
| oYo1380 | CACCTTTACTCACGGCGGCCGCCATCACCGCAGCAATGC |
| oYo1381 | TGGGCTAGCGAATTCGAGCTATGCCGAAATGGATTCTATGC |
| oYo1382 | CACCTTTACTCACGGCGGCCGCCTTCACACACACCACATTG |
| oYo1383 | TGGGCTAGCGAATTCGAGCTATGAAAATCGCTATGATTGGTC |
| oYo1384 | CACCTTTACTCACGGCGGCCGCGCCCTTGGTTACCTGTTG |
| oYo1385 | TGGGCTAGCGAATTCGAGCTATGAAACCTATGCAGCGTC |
| oYo1386 | CACCTTTACTCACGGCGGCCGCTTGTTGATTAACAAAGGCTTTA |
| oYo1387 | TGGGCTAGCGAATTCGAGCTATGTCTTTCCCTGTTCTCATC |
| oYo1388 | CACCTTTACTCACGGCGGCCGCGAAGTCCTCCATTAAATAGGAAAG |
| oYo1393 | CCGCATGCGATATCGAGCTCTCCCTCAGTGATGAACAGCAGTTC |
| oYo1394 | CTTTATCAATAAATGATTCAGCGGTAGGCCTTATGTTCTATGATGC |
| oYo1395 | GCATCATAGAACATAAGGCCTACCGCTGAATCATTTATTGATAAAG |
| oYo1396 | CGGATAACAATTTGTGGAATTCCCCGGAATAGCATAAATCGGTG |
| oYo1397 | CCGCATGCGATATCGAGCTCTCCCGTTATTCTGGTGATTGATGTTTGT |
| oYo1398 | CTTAAGGAAACGAAGCGATTGGGCCACATTGTTTAACCTAC |
| oYo1399 | GTAGGTTAAACAATGTGGCCCAATCGCTTCGTTTCCTTAAG |
| oYo1400 | CGGATAACAATTTGTGGAATTCCCGATGATGAGCAATCGCC |
| YPR347 | GCTCTAGAGAGGGCGATATTGATTATCC |
| YPR348 | CCTAGCTAGCTCTTAAATCGTGAGCGTTTC |
| YPR351 | CCTAGCTAGCGTGGCACGTCTACTTGGTGAAC |
| YPR352 | GCTCTAGATTAAGTCCTAGTCAAACGCGCG |
| pBAD_F | ATGCCATAGCATTTTTATCC |
| pBAD_R | GATTTAATCTGTATCAGG |
