# Supplementary figures and images for: Analysis of HubP-dependent cell pole protein targeting in Vibrio cholerae uncovers novel motility regulators
Source: PLoS Genet. 2022 Jan 12;18(1):e1009991. doi: 10.1371/journal.pgen.1009991 (PMC8789113; doi:10.1371/journal.pgen.1009991)

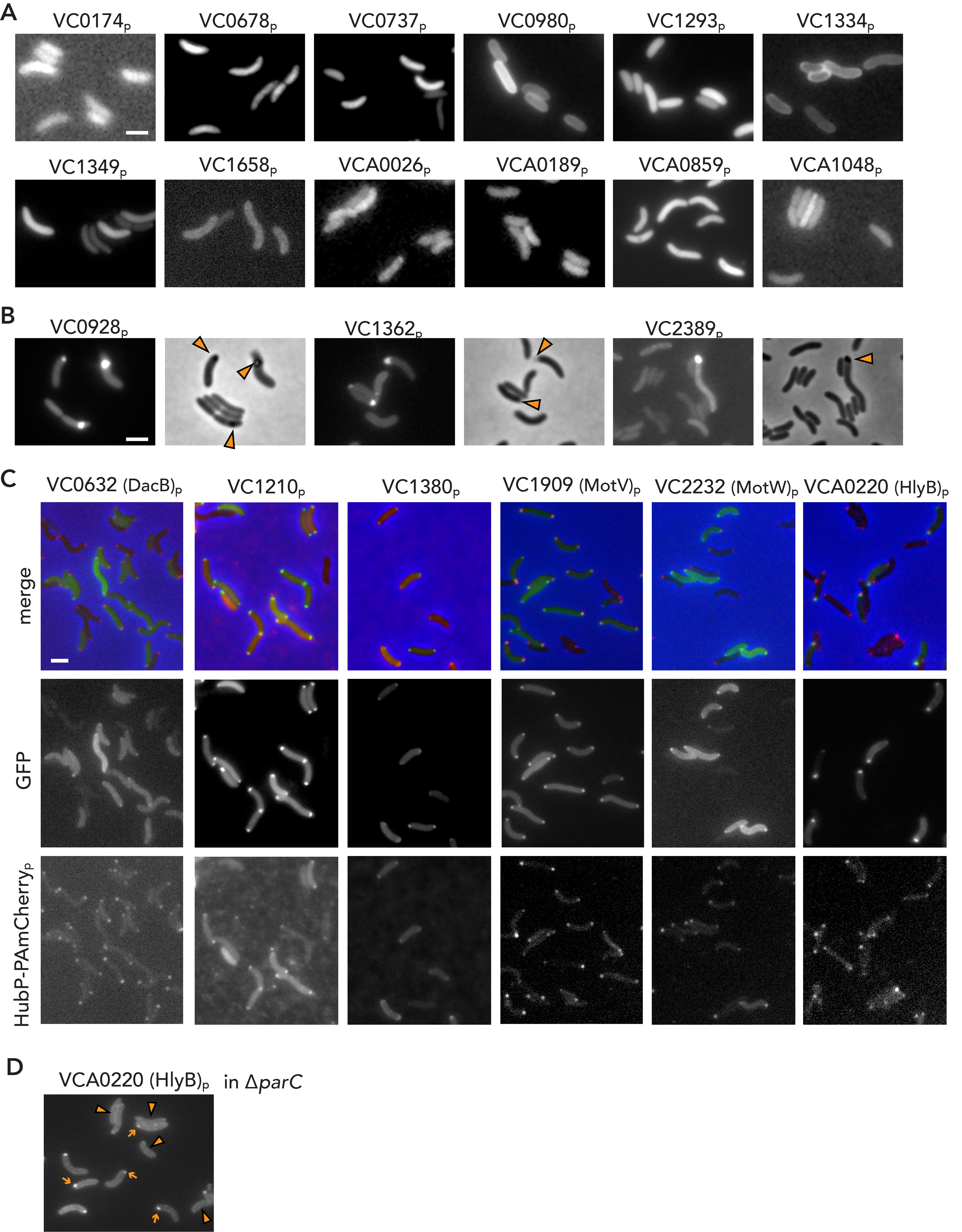

Supplement: S1 Fig — Representative fluorescent microscopy images of C-terminal GFP fusions of candidate polar proteins in WT V. cholerae. p, plasmid-based expression. (A) Protein fusions showing diffuse distribution. (B) Protein fusions showing inclusion body formation. Inclusion bodies observed in the corresponding phase contrast images are indicated with arrowheads. Protein fusions exhibiting polar foci are shown in main Fig 1C. (C) Representative fluorescence microscopy images of GFP fusions of identified polar proteins (pseudocolored in green), coexpressed with HubP-PAmCherry (pseudocolored in red). Phase contrast images are also shown in blue. (D) VCA0220-GFP localization in ΔparC V. cholerae cells. Arrowheads indicate cells with diffuse fluorescence and without foci; arrows indicate slightly misplaced foci. Bars = 2 μm. (TIF) [file pgen.1009991.s006.tif]

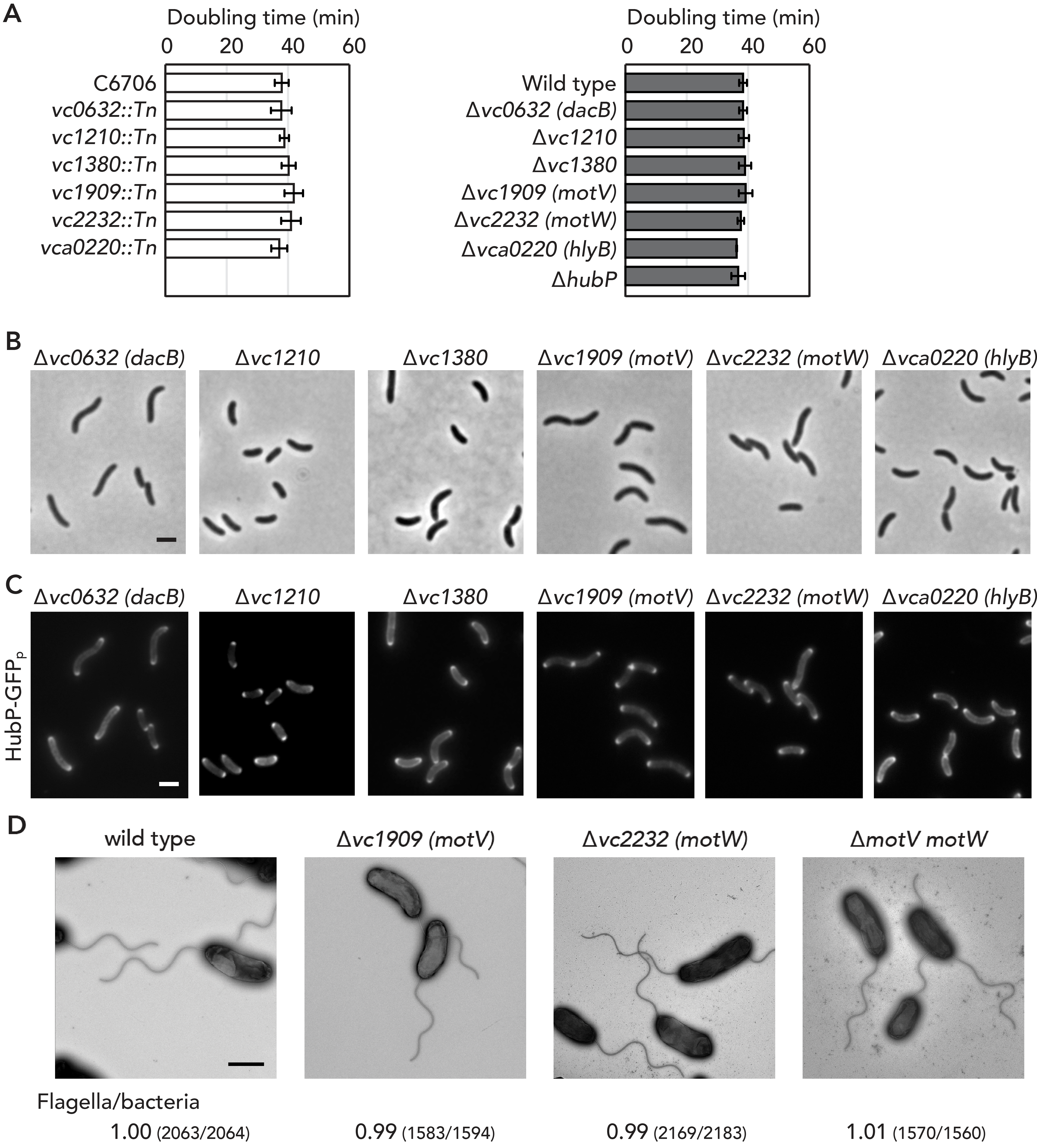

Supplement: S2 Fig — (A) Growth rate measured by microplate reader. White bars in the left panel indicated transposon insertion mutants obtained from the defined V. cholerae transposon mutant library [31], derived from the C6706 strain. Grey bars in the right panel indicate clean in-frame deletion mutants obtained from the N16961 WT strain. Average and standard deviations of at least 3 independent experiments are shown. (B) Representative phase contrast images of indicated mutants. (C) Representative fluorescent microscopy images of HubP-GFP expressed from the plasmid in indicated mutants. (D) Representative negative-stain transmission electron microscopy images of indicated mutants. Numbers of flagella and bacteria detected in images were shown below. Bars, 2 μm (fluorescence microscopy); 1 μm (TEM). (TIF) [file pgen.1009991.s007.tif]

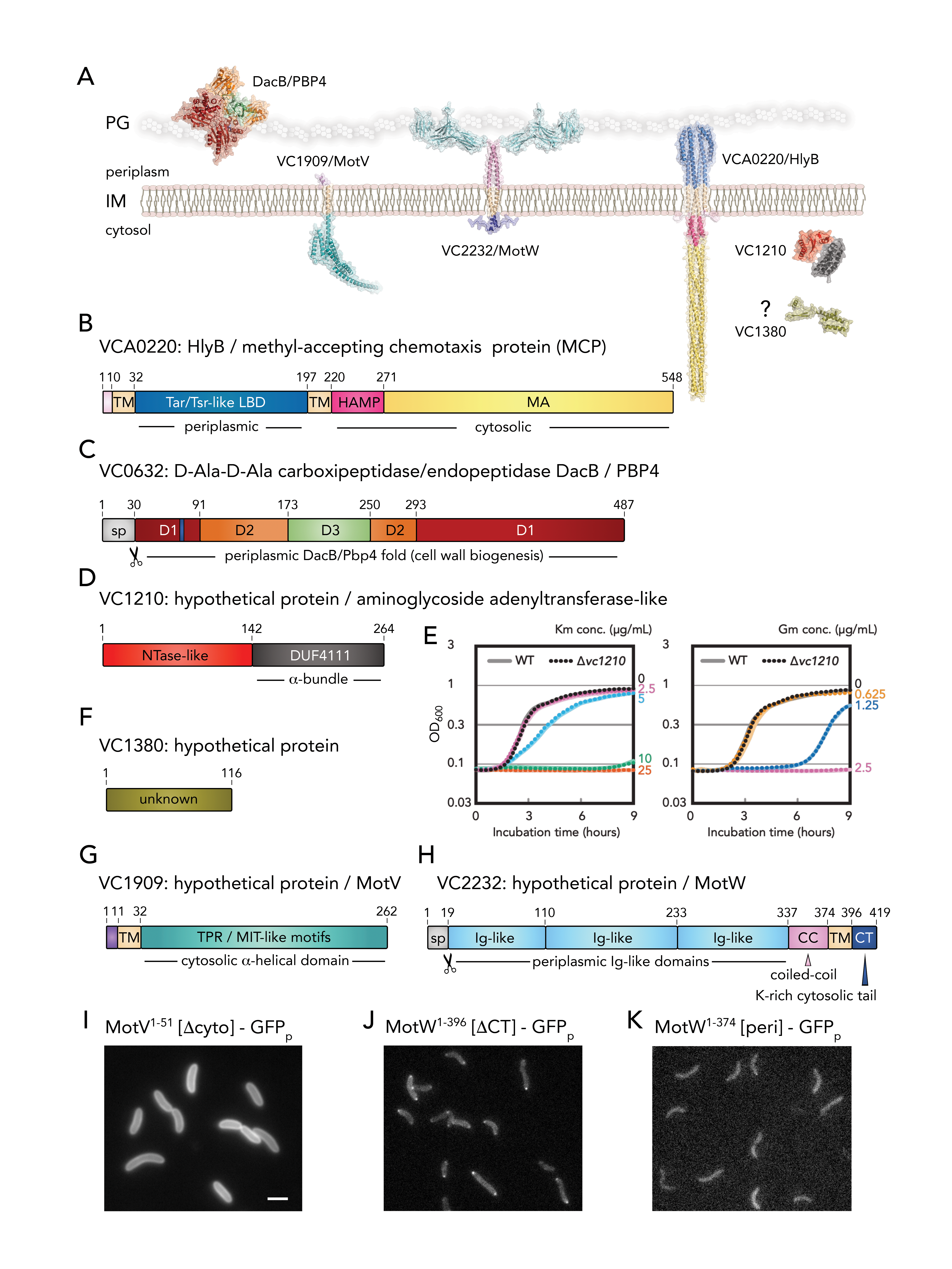

Supplement: S3 Fig — (A) Predicted protein folds and topologies. IM, inner membrane; PG, peptidoglycan. Protein fold prediction was carried out with Phyre2, Robetta, and AlphaFold2 and consensus protein folds were visualized with PyMOL (Schrödinger, LLC). Signal peptide detection was carried out with the SignalP-5.0 server. VC1210 and VC1380 do not feature detectable signal peptides for secretion or inner membrane targeting. (B) Predicted VCA0220/HlyB domain architecture. TM, transmembrane region; Tar, taxis towards aspartate; Tsr, taxis towards serine; LBD, ligand-binding domain; HAMP, histidine kinase-adenylate cyclase-methyl accepting protein-phosphatase domain; MA: methyl-accepting domain. Protein dimerization is based on multiple structures of conserved MCP proteins. (C) Predicted VC0632/DacB/PBP4 domain architecture. sp, signal peptide; D, domain. Protein dimerization is based on multiple structures of conserved DacB homologs. (D) Predicted VC1210 domain architecture. NTase-like, nucleotydiltransferase-like domain sharing predicted structural homology with the NTase domains of aminoglycoside adenyltransferases; DUF4111: α-helical domain of unknown function DUF4111/PF13427. (E) Representative growth curves of WT and Δvc1210 V. cholerae in the presence of the indicated concentrations of antibiotics. Km, kanamycin; Gm, gentamycin. OD600, optical density upon detection of transmitted light with 600 nm wavelength. Results are representative of three independent experiments. (F) VC1380 domain architecture. Primary sequence and fold prediction analyses do not detect conserved domains with significant confidence. (G) Predicted VC1909/MotV architecture. TPR, tetratricopeptide repeat; MIT, microtubule interacting and trafficking. (H) Predicted VC2232/MotW architecture. Ig, immunoglobulin; CC, coiled-coil; CT, C-terminus; K-rich, lysine-rich. Proposed protein dimerization is based on the presence of predicted coiled-coil regions in the protein, as well as on bacterial two-hybrid (BACTH) comp [file pgen.1009991.s008.tif]

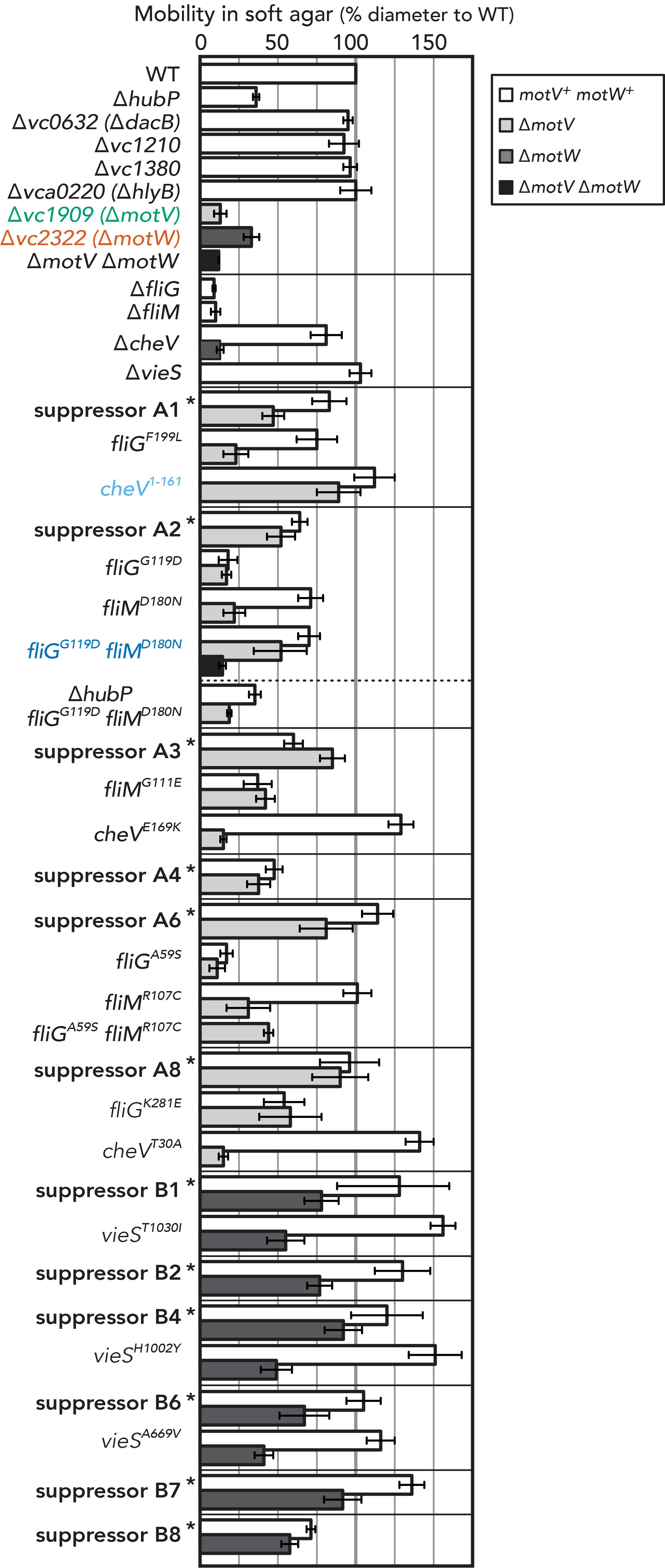

Supplement: S5 Fig — Complete graphs for motility defects examined by swimming in soft agar plates. Average diameter relative to WT, along with standard deviations from three or more experiments are shown. ΔmotV and/or ΔmotW strains are indicated in colored bars and motV+ motW+ strains are indicated in blank bars. * motV+ complemented in the suppressor strain. (TIF) [file pgen.1009991.s010.tif]
